# Predictive modeling of multi-class diabetes mellitus using machine learning and filtering iraqi diabetes data dynamics

**DOI:** 10.1371/journal.pone.0300785

**Published:** 2024-05-16

**Authors:** Md Abdus Sahid, Mozaddid Ul Hoque Babar, Md Palash Uddin

**Affiliations:** Department of Computer Science and Engineering, Hajee Mohammad Danesh Science and Technology University, Dinajpur, Bangladesh; St Xavier’s Catholic College of Engineering, INDIA

## Abstract

Diabetes is a persistent metabolic disorder linked to elevated levels of blood glucose, commonly referred to as blood sugar. This condition can have detrimental effects on the heart, blood vessels, eyes, kidneys, and nerves as time passes. It is a chronic ailment that arises when the body fails to produce enough insulin or is unable to effectively use the insulin it produces. When diabetes is not properly managed, it often leads to hyperglycemia, a condition characterized by elevated blood sugar levels or impaired glucose tolerance. This can result in significant harm to various body systems, including the nerves and blood vessels. In this paper, we propose a multiclass diabetes mellitus detection and classification approach using an extremely imbalanced Laboratory of Medical City Hospital data dynamics. We also formulate a new dataset that is moderately imbalanced based on the Laboratory of Medical City Hospital data dynamics. To correctly identify the multiclass diabetes mellitus, we employ three machine learning classifiers namely support vector machine, logistic regression, and k-nearest neighbor. We also focus on dimensionality reduction (feature selection—filter, wrapper, and embedded method) to prune the unnecessary features and to scale up the classification performance. To optimize the classification performance of classifiers, we tune the model by hyperparameter optimization with 10-fold grid search cross-validation. In the case of the original extremely imbalanced dataset with 70:30 partition and support vector machine classifier, we achieved maximum accuracy of 0.964, precision of 0.968, recall of 0.964, F1-score of 0.962, Cohen kappa of 0.835, and AUC of 0.99 by using top 4 feature according to filter method. By using the top 9 features according to wrapper-based sequential feature selection, the k-nearest neighbor provides an accuracy of 0.935 and 1.0 for the other performance metrics. For our created moderately imbalanced dataset with an 80:20 partition, the SVM classifier achieves a maximum accuracy of 0.938, and 1.0 for other performance metrics. For the multiclass diabetes mellitus detection and classification, our experiments outperformed conducted research based on the Laboratory of Medical City Hospital data dynamics.

## Introduction

Diabetes is a chronic metabolic disease associated with high blood glucose (also known as blood sugar), which can adversely affect the heart, blood vessels, eyes, kidneys, and nerves over time. It is a long-term condition that develops when the body either produces insufficient insulin or cannot properly utilize the insulin produced. Insulin is a hormone that controls blood sugar levels. Uncontrolled diabetes frequently causes hyperglycemia, also known as impaired glucose tolerance or raised blood sugar, which can severely damage many different bodily systems, including the neurons and blood vessels. According to the IDF Diabetes Atlas Tenth Edition 2021, 537 million individuals (20-79 years) have diabetes [1]. Adults with diabetes make up three out of four inhabitants of low- and middle-income nations. By 2045, it is anticipated that 783 million people worldwide will be affected by diabetes.

People with diabetes are more likely to suffer severe illness and die from other diseases. The highest number of adults and older people living in lower and middle-income countries are highly affected by this fatal disease [2, 3]. Since treatment can be expensive and often results in significant personal costs, the disease becomes a significant burden on the regular lives of civilians. Additionally, diabetes accounted for 9% of all adult healthcare expenditures, amounting to at least US $966 billion. To reduce the chance of death and control blood sugar levels, early detection or diagnosis of diabetes is essential [4]. Undiagnosed diabetes may cause complications such as diabetic retinopathy, nephropathy, neuropathy, stroke, and foot ulcers [5]. Therefore, it is crucial to detect diabetes early in order to improve patient’s quality of life and lengthen their lives [6]. The basic two types of diabetes are juvenile diabetes (Type-I) and adult-onset diabetes (Type-II). The typical ways it appears include pre-diabetes, which is characterized by higher-than-normal blood glucose levels, overt type I and type II diabetes [7, 8], and gestational diabetes, which occurs during pregnancy [9]. However, racial differences in the glycemic threshold levels for diabetes detection may exist due to variations in glycemic risk levels among various ethnic groups [10]. Type I diabetes is a hereditary disease that frequently appears in adolescence. Obesity and adult-onset diabetes are linked, although both can be prevented or managed with a healthy diet and regular exercise [11]. Other types of diabetes include gestational diabetes and pre-diabetes. Gestational diabetes is first diagnosed during pregnancy, while pre-diabetes means that blood sugar levels are higher than normal but not high enough to be considered type 2 diabetes [12]. In this study, we provide a wide-ranging empirical analysis spanning many popular ML classifiers on diabetes disease in the case of multiclass classification, namely healthy control, pre-diabetes, and diabetes prediction [13]. The main contributions of this study are threefold:

- This paper primarily focused on the generation of a new multiclass moderately imbalanced LMCH data dynamics from the existing extremely imbalanced LMCH data dynamics. Significant attention has been paid to developing filtered moderately imbalanced LMCH data dynamics.
- Rigorous and empirical analysis for the classification of diabetes disease with hyperparameter optimization.
- In the context of feature analysis, we employ filter methods (Information Gain), wrapper methods (Sequential Feature Selection, Backward Feature Elimination), and an embedded method (Random Forest), while also assessing the significance of these methods by considering classification performance.

## Literature review

In the preceding study, a variety of methods for diabetes prediction were applied. In our investigation, we looked at recent scholarly works from the past eight years, specifically from 2015 to 2022. These are summarized below:

Chollette et al. [10], 2022, developed a machine learning framework for diabetes prediction and diagnosis using the benchmark PIMA Indian dataset and the Laboratory of the Medical City Hospital (LMCH) diabetes dataset. The framework adopted polynomial regression and Spearman correlation for feature selection and missing value imputation to enhance performance. Their study used both datasets to classify and forecast diabetes using the random forest (RF), support vector machine (SVM), and twice-growth deep neural network (2GDNN) models. The 2GDNN model achieved 97.34% precision, 97.24% sensitivity, 97.26% F1-score, 99.01% train-accuracy, and 97.25% test-accuracy, demonstrating 97.931% and 100% classification accuracy attained on the PIMA Indian dataset. Similar results were obtained on the LMCH dataset, with an accuracy of 97.333%. Usama Ahmed et al. [14], 2022, presented a fused machine learning model FMDP for diabetes prediction using the PIMA Indian Diabetes Dataset (PIDD). They divided the preprocessing dataset into a 70:30 ratio for training and testing. Two state-of-the-art algorithms, namely SVM and ANN, were used, and the outcome of these models was then used as input for the fuzzy model. Finally, the fuzzy model was utilized for diabetes prediction. In the testing phase, SVM achieved 89.1% accuracy, 89.39% recall, 88.89% specificity, and 93.42% F1-score, while ANN achieved 92.31% accuracy, 93.85% recall, 91.21% specificity, and 94.12% F1-score. The proposed FMDP model achieved 94.87% accuracy, 95.52% recall, 94.38% specificity, and 94.12% F1-score, which was comparatively higher than other models. Himanshu Gupta et al. [15], 2022, compared the performance of deep learning (DL) and quantum machine learning (QML) algorithms for the prediction of diabetes using the benchmark PIDD dataset. Their preprocessed dataset was fed as input to these classifiers. Using DL algorithms, they achieved 95% accuracy, 90% precision, 95% recall, and 93% F1-score. The QML algorithms provided 86% accuracy, 74% precision, 85% recall, and 79% F1-score. Muhammad Mazhar et al. [16], 2021, developed a more effective ANN model for diabetes prediction using the artificial backpropagation scaled conjugate gradient neural network (ABP-SCGNN) algorithm. They used the benchmark PIDD and assessed the performance using the mean squared error (MSE) and accuracy measures on a substantial dataset. The ABP-SCGNN model outperformed other ANN models in terms of accuracy, achieving 93% on the validation set. Pratya et al. [17], 2021, suggest an innovative prediction technique called Average Weighted Objective Distance (AWOD). This technique is based on the presumption that individuals have a variety of health issues caused by various personal circumstances, highlighting the need for an effective prediction model. The study utilizes two datasets to validate the proposed method: the benchmark PIMA Indian Dataset and the LMCH Dataset, which is labeled by the PIDD data to balance each individual dataset. The datasets are split into a 70-30 ratio. Machine learning methods are employed to compare the evaluation of the two datasets. Comparison is made using AWOD weighted values and predicted classes to ensure accurate predictions. The weight values measure both significant and insignificant factors. The proposed method achieves an accuracy of 93.22% in Dataset 1 and 98.85% in Dataset 2. Kuang-Ming Kuo et al. [18], 2020, built a predictive model based on numerous machine learning algorithms and ensemble algorithms on a large dataset of 149 patients who have contracted type II diabetes mellitus. They also compared the adopted machine learning algorithms and included features with state-of-the-art works. They obtained the best performance by adopting a 10-fold CV, leave-one-subject-out, and holding-out strategy, with SVM and RF revealing the best classification metrics. The information gain and gain ratio discriminated the features and ranked them up. They achieved the highest accuracy of 100% with SVM and RF, along with other evaluation metrics such as AUC, MCC, Precision, Recall, and F1-score, all achieving 100%. Md. Kamrul Hasan et al. [19], 2020, proposed a diabetes prediction framework using the benchmark PIDD dataset. In this research work, they proposed an ensemble learning, AB+XB, algorithm and compared the result with some state-of-the-art algorithms. In the data preprocessing phase, they employed different types of techniques to convert the raw data to trainable data. They also used three feature selection techniques, namely PCA, independent component analysis (ICA), and correlation-based technique. Their proposed AB+XB ensemble model achieved 78.9% sensitivity, 93.4% specificity, 92% false omission rate, 66.23% diagnostic odd ratio, and 95% AUC. They claimed that their proposed ensemble model outperformed the state-of-the-art algorithms in terms of AUC. Tuan Minh Le et al. [20], 2020, proposed feature selection-based early-stage diabetes prediction. They used a diabetes dataset collected from a hospital in Sylhet, Bangladesh. They mainly used MLP for prediction, along with state-of-the-art machine learning algorithms for comparison purposes. They applied two feature selection algorithms, namely grey wolf optimization (GWO) and adaptive particle grey wolf optimization (APGWO). Using GWO techniques, they selected 13 out of 15 features and achieved 96% accuracy, 100% precision, 93% recall, and 97% F1-score for MLP. They also selected 9 out of 15 features and achieved 97% accuracy, 99% precision, 97% recall, and 98% F1-score for MLP. The prediction performance of the APGWO-MLP model was superior to the other models used for comparison. K. Vidhya et al. [21], 2020, proposed diabetes prediction based on deep belief network (DBN). They preprocessed the dataset using traditional preprocessing techniques. Their proposed DBN model achieved an accuracy of 81.2%. In comparison, SVM, RF, LR, and CNN achieved accuracies of 80.1%, 80.07%, 80.11%, and 81.02%, respectively. Huaping Zhou et al. [22], 2020, proposed a DL-based diabetes prediction. In this research paper, they employed two datasets, namely the benchmark PIDD and the diabetes type dataset (DTD) from Data World. Their proposed model consisted of several layers, and to avoid the problem of overfitting, they added a regularization layer. Their model also used a batch normalization layer. They claimed that the DTD dataset provided a score of 94.02%, and the PIDD dataset provided a score of 99.41%. Huma Naz et al. [23], 2020, presented a DL-based approach for diabetes prediction. They evaluated their methods on the benchmark PIDD dataset and compared their proposed DL model against the artificial neural network (ANN), decision tree (DT), and Naive Bayes (NB) models. Their proposed DL model is a multilayer feed-forward perceptron-based model that enhances the characteristics of ANN and is trained using back-propagation stochastic gradient descent. The NB models achieved 76.33% accuracy, 59.07% precision, 64.51% recall, 61.67% F1-score, and 84.29% specificity. The DT models achieved 96.62% accuracy, 94.02% precision, 95.45% recall, 94.72% F1-score, and 97.86% specificity. On the other hand, the ANN achieved 90.34% accuracy, 88.05% precision, 83.09% recall, 85.98% F1-score, and 91.43% specificity. Their proposed DL models showed the highest performance with 98.07% accuracy, 95.22% precision, 98.46% recall, 96.81% F1-score, and 99.29% specificity. Hang Lai et al. [24], 2019, presented a machine learning-based diabetes prediction study. They conducted their research using a dataset collected from the Canadian Primary Care Sentinel Surveillance Network (CPCSSN). Their main goal was to develop a model that would provide high performance in identifying diabetes in Canadian individuals. For this purpose, they used logistic regression (LR) and gradient boosting (GB) algorithms. The LR models achieved 71.6% sensitivity, 82.3% specificity, 19.6% misclassification rate (MCR), and 84% ROC score. The GB models achieved 48.3% sensitivity, 95.2% specificity, 15% MCR, and 84.7% ROC score. Qian Wang et al. [25], proposed a diabetes mellitus classification method named DMP_MI. They used a benchmark PIDD dataset and handled missing values using Naïve Bayes (NB) methods. They applied an oversampling method called adaptive synthetic (ADASYN) to balance the dataset. They evaluated their method against RF, NB, SVM, and DT models using 5-fold and 10-fold cross-validation (CV). In the case of 5-fold CV, DMP_MI achieved 87.1% accuracy, 80.6% precision, 85.4% recall, 83% F1-score, and 92.8% AUC. RF achieved 78.6% accuracy, 73.3% precision, 63% recall, 67.3% F1-score, and 87% AUC. In the case of 10-fold CV, DMP_MI achieved 86.2% accuracy, 78.5% precision, 85.7% recall, 81.6% F1-score, and 92.6% AUC. RF achieved 77.9% accuracy, 68.8% precision, 75.9% recall, 72.1% F1-score, and 84.1% AUC. For NB, they achieved 76.3% accuracy, 75.9% precision, 76.3% recall, 76% F1-score, and 81.9% AUC. SVM achieved 65.1% accuracy, 42.4% precision, 65.1% recall, 51.3% F1-score, and 50% AUC. DT achieved 73.8%, 73.5%, 73.8%, 73.6%, and 75.1% for accuracy, precision, recall, F1-score, and AUC, respectively. Pei et al. [26], evaluated the performance of five well-known classifiers (J48, AdaboostM1, SMO, Bayes Net, and Naive Bayes) in detecting people with diabetes using 9 clinical features. They applied machine learning and data mining techniques to assess the efficiency of these classifiers in quickly and accurately identifying individuals with diabetes. They also compared the characteristics of different features between diabetic and non-diabetic individuals. The DT produced by the J48 algorithm achieved an accuracy of 95.03%, precision of 95%, recall of 95%, F1-score of 94.8%, and AUC of 96.4%, making it the best classifier. Talaei-Khoei et al. [27], 2018, assessed the effectiveness of classification algorithms in identifying patients at risk of developing Type 2 diabetes (T2D) in the short, medium, and long terms. Their study obtained 10,911 records from 36 clinics and employed syntactic minority oversampling and random undersampling to establish a balanced dataset. They evaluated different machine learning approaches for T2D development identification using AUC, sensitivity, specificity, Matthew Correlation Coefficient, and Mean Calibration Error. Md. Maniruzzaman et al. [28], 2018, presented a machine learning-based diabetes prediction using the benchmark PIDD dataset. They applied six feature selection techniques, namely RF, LR, mutual information, PCA, ANOVA, and Fisher Discriminant Ratio (FDR), on the preprocessed dataset. They used several algorithms to predict diabetes and achieved the best performance using RF classification with RF feature selection, with accuracy, sensitivity, specificity, and AUC of 92.26%, 95.96%, 79.72%, and 93%, respectively. Alghamdi et al. [29], 2017, built an ensembling-based prediction model using multiple linear regression, information gain ranking, and 13 clinically significant features. They used the Synthetic Minority Oversampling Technique (SMOTE) to mitigate the effects of class imbalance. They achieved an accuracy prediction of 92% by combining three DT models in the ensembling Vote approach. Nilashi et al. [30], 2017, developed an intelligent system for classifying diabetes disease using machine learning approaches. They used the PIMA Aboriginals diabetes dataset provided by NIDDK and John Hopkins University. They employed self-organizing maps (SOM), and neural networks (NN) for clustering, PCA, noise removal, and classification tasks. They compared their proposed method with other classifiers and achieved an accuracy of 92.28%. Esteban et al. [31], 2017, compared the effectiveness of multiple classification algorithms in classifying patients based on their diabetic status using data from electronic health records (EHRs). The stacked generalization meta-learner performed the best with a Kappa coefficient value of 0.95 (95% CI 0.91, 0.98). Md. Maniruzzaman et al. [32], 2017, proposed a gaussian process classifier (GPC)-based diabetes mellitus classification using the PIDD dataset. They compared their proposed method with linear discriminant analysis (LDA), quadratic discriminant analysis (QDA), and NB. They applied 5-fold and 10-fold CV. Using 5-fold CV, GPC achieved 81.44% accuracy, 88.65% sensitivity, and 62.04% specificity, which were the highest scores compared to LDA, QDA, and NB. With 10-fold CV, GPC achieved 81.97%, 91.79%, and 63.33% accuracy, sensitivity, and specificity, respectively, which were also the highest scores compared to LDA, QDA, NB, and 5-fold CV. N. Yuvaraj et al. [33], 2017, introduced diabetes prediction based on machine learning on a Hadoop cluster. They used the benchmark PIDD dataset for evaluation. Their approach utilized 7 out of 8 independent features and employed RF, NB, and DT models. They achieved 94%, 94%, 88%, and 91% accuracy, precision, recall, and F1-score, respectively, using the RF models. The NB models provided 91%, 91%, 82%, and 86% accuracy, precision, recall, and F1-score, respectively. The DT models achieved 88% accuracy, 87% precision, 77% recall, and 82% F1-score. Kagawa et al. [34], 2016, presented a practical phenotyping framework that combines expert knowledge and a machine learning approach to generate two phenotyping algorithms for screening and selecting study subjects. Binary classification was used to ascertain whether a patient has T2D. They introduced two new evaluation metrics, AUPS with high sensitivity and AUPS with high positive predictive value. Anderson et al. [35], 2015, constructed prediction model ensembles for progression to prediabetes or T2D using an aggregated EHR data sample and a novel analytical platform called Reverse Engineering and Forward Simulation. They analyzed data from 24,331 individuals between 2007 and 2012 to predict transitions to prediabetes or T2D. The primary ensemble of models accurately predicted progression to T2D with an AUC of 0.76, incorporating established risk factors. Models for progression to prediabetes included novel factors and achieved an AUC of 0.70.

After reviewing the aforementioned research papers, we have reached the conclusion that the prediction of diabetes has been approached in various ways across different studies. It is noteworthy that most of these papers do not specifically address the prediction of prediabetes, which is an important stage in the development of diabetes. While some papers focus solely on predicting the onset of diabetes, others may not explicitly mention the prediction of prediabetes due to factors such as data availability or the specific research objectives of the authors. However, it is crucial to recognize the significance of predicting prediabetes, as it provides an opportunity for early intervention and preventive measures to delay or even prevent the onset of diabetes.

## Materials and methods

**Fig 1:**
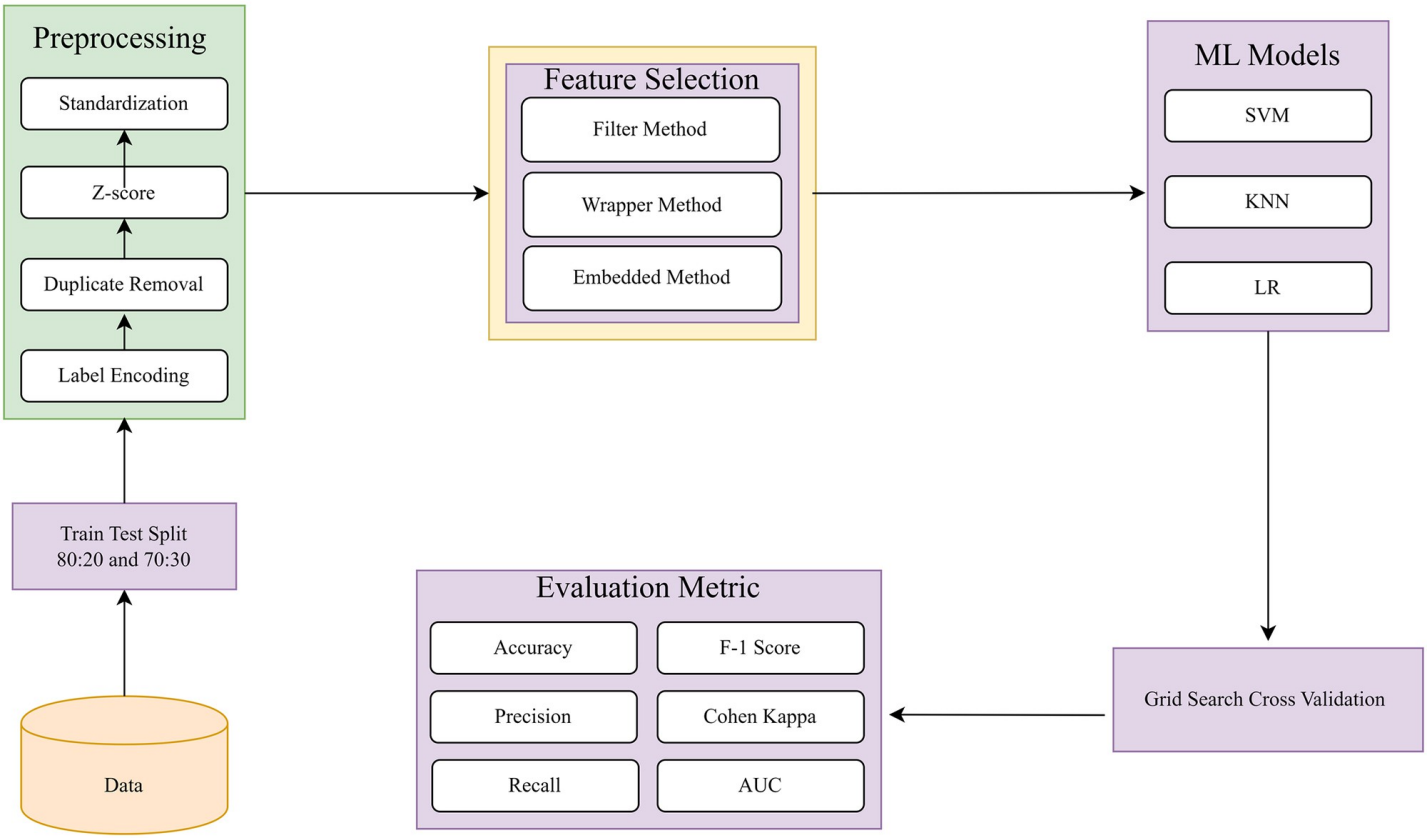
Proposed methodology for diabetes mellitus prediction includes several steps, including data preprocessing and feature selection.

In this section, we will assess the data features that are essential for predicting diabetes. We will analyze the key components of the methodology used in this study, which include: (i) dataset preprocessing, (ii) feature selection, (iii) training the machine learning classifier and hyperparameter optimization, and (iv) performance evaluation. A visual representation of the proposed methodology is presented in Fig 1.

### Dataset description and creating new filtered dataset

This dataset contains diabetes-related information on Iraqi individuals and was provided by the Laboratory of Medical City Hospital (LMCH), Iraq. The LMCH dataset [36] consists of a total of 14 features. The dataset includes three prediction classes: diabetes negative, prediabetes, and diabetes positive, with a total of 1000 instances. Table 1 provides an overview of the original LMCH diabetes dataset, which exhibits an extremely imbalanced class distribution. Due to this extreme imbalance, we created a new dataset from the LMCH dataset with a moderately imbalanced class distribution, as shown in the table, in order to address the issue of class imbalance.

**Table 1:** Overview of the original extremely imbalance LMCH and our created filtered moderately imbalance LMCH diabetes data dynamics.

					Original LMCH			Filtered LMCH^a^		
SN	Feature	Description	Type	Value	Positive Classes (%)	Negative Classes (%)	Pre Diabetes Classes (%)	Positive Classes (%)	Negative Classes (%)	Pre Diabetes Classes (%)
1	Gender	1 = Male; 0 = Female	Categorical	0 / 1		103 (10.3%)	53 (5.3%)	128 (48.48%)	96 (36.36%)	40 (15.15%)
2	AGE	Age measured in years	Numerical	20–79						
3	Urea	Urea (mg/dl)	Numerical	0.5–38.9						
4	Cr	Creatinine ratio (μmol/L)	Numerical	6–800						
5	HbA1c	Hemoglobin A1c (mmol/L)	Numerical	0.9–16						
6	Chol	Cholesterol (mmol/L)	Numerical	0–10.3	844					
7	TG	Triglyceride (mmol/L)	Numerical	0.3–13.8	(84.4%)					
8	HDL	High Density Lipoprotein (mmol/L)	Numerical	0.2–9.9						
9	LDL	Low Density Lipoprotein (mmol/L)	Numerical	0.3–9.9						
10	VLDL	Very Low Density Lipoprotein (mmol/L)	Numerical	0.1–35						
11	BMI	Body Mass Index (Kg/Sq. m)	Numerical	19–47.75						
12	Class	0 = Diabetes absent	Categorical	0, 1 and 2						
		1 = Pre diabetes								
		2 = Diabetes present								

^*a*^Description of Dataset Creation: After removing duplicate instances from extremely imbalance data dynamics, there are total 826 unique instances (pre-diabetes = 40, healthy control = 96, and diabetes-positive = 690. For the moderately imbalanced LMCH data dynamics, we keep the instances of prediabetes = 40 and healthy control = 96. In the case of diabetes-positive instances, we calculate the mean of features. Then, we filter out the instances based on the mean. For example, if 11.32, 11.24, 11.3, and 10.95 represent the mean of features then we keep one of them as a representative of others. In this way, we selected diabetes-positive instances for the moderately imbalanced LMCH data dynamics. The moderately imbalanced LMCH data dynamics contain a total of 264 unique instances of three types of classes (pre-diabetes, diabetes, and healthy control). Check out our created dataset in [37]

### Dataset analysis and visualization insights

This section provides detailed descriptions and visualizations of the data.

#### Statistical description

Table 2 provides the descriptive statistics for each feature, including the minimum, maximum, mean, and standard deviation scores of the original extremely imbalanced LMCH data dynamics and our filtered moderately imbalanced LMCH data dynamics.

**Table 2:** Statistical characteristics of the original extremely imbalance LMCH and filtered moderately imbalance LMCH diabetes data dynamics.

Feature	Original LMCH				Filtered LMCH			
	Min	Max	Mean	Std	Min	Max	Mean	Std
Gender	0.0	1.0	0.565	0.496	0.0	1.0	0.545	0.499
AGE	20.0	79.0	53.528	8.799	25.0	77.0	49.523	10.127
Urea	0.5	38.9	5.125	2.935	1.1	26.4	5.672	4.003
Cr	6.0	800.0	68.943	59.985	6.0	800.0	85.807	99.4
HbA1c	0.9	16.0	8.281	2.534	0.9	14.6	6.863	2.545
Chol	0.0	10.3	4.863	1.302	0.0	9.5	4.594	1.289
TG	0.3	13.8	2.35	1.401	0.6	8.7	2.152	1.266
HDL	0.2	9.9	1.205	0.66	0.4	4.0	1.183	0.456
LDL	0.3	9.9	2.61	1.115	0.3	5.6	2.531	1.0
VLDL	0.1	35.0	1.855	3.664	0.2	31.8	1.479	3.1
BMI	19.0	47.75	29.578	4.962	19.0	43.25	26.627	5.094
Class	0.0	2.0	1.741	0.631	0.0	2.0	1.121	0.915

#### Feature prevalence

**Fig 2:**
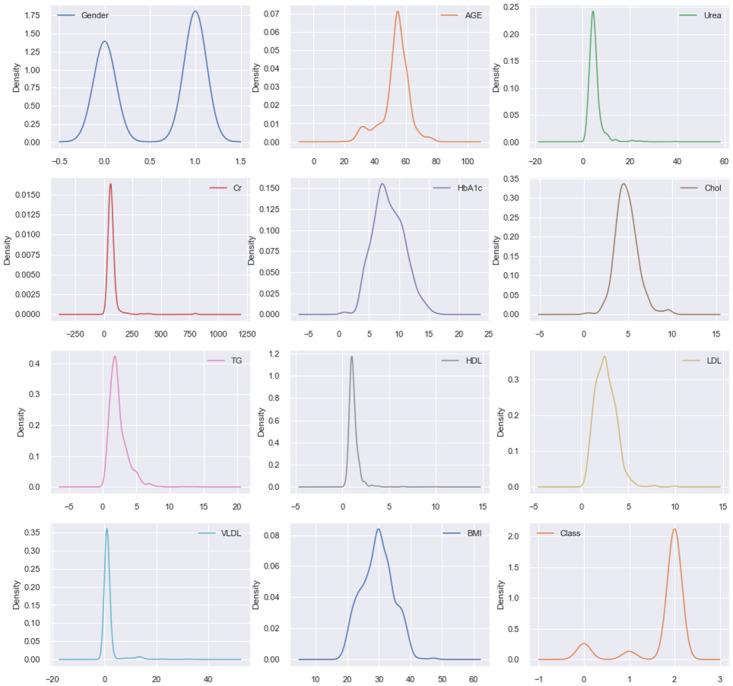
The KDE plot of the original extremely imbalanced LMCH data dynamics.

**Fig 3:**
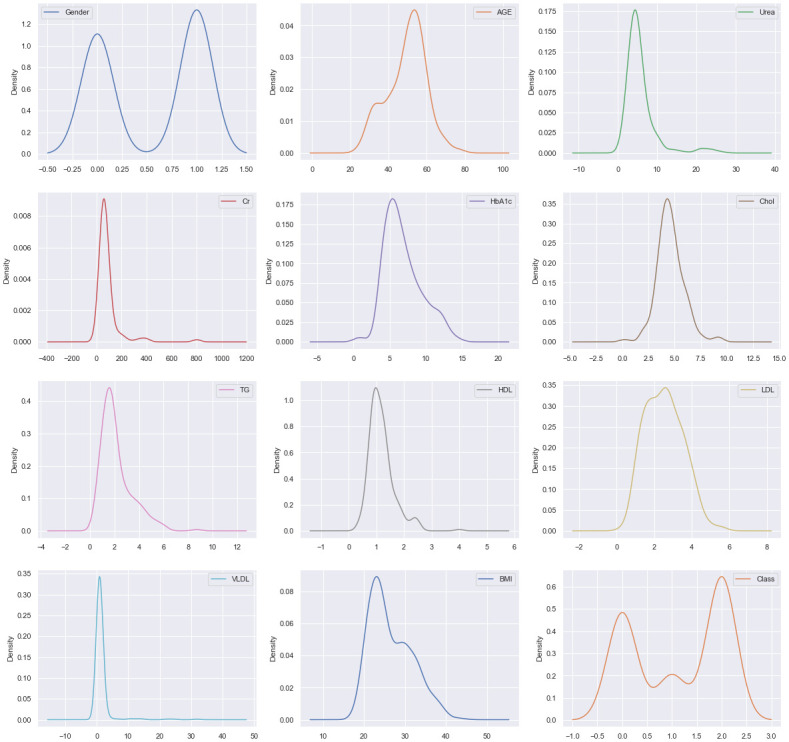
The KDE plot of the filtered moderately imbalanced LMCH data dynamics.

Figs 2 and 3 represent the Kernel Density Estimation (KDE) plot for the experimental dataset, which is a popular technique for estimating the probability distribution of data. The KDE plot is created by generating a small consistent curve (also known as a kernel) for each independent data point along an axis and then adding all of these curves together to form a single smooth density estimation. The KDE plot for the experimental data dynamics shows a normal distribution or Gaussian distribution for all the features. From Fig 3, it is evident that the KDE plot of our created filtered LMCH data dynamics closely resembles that of the original LMCH data dynamics.

#### Pearson-correlation heatmap visualization

**Fig 4:**
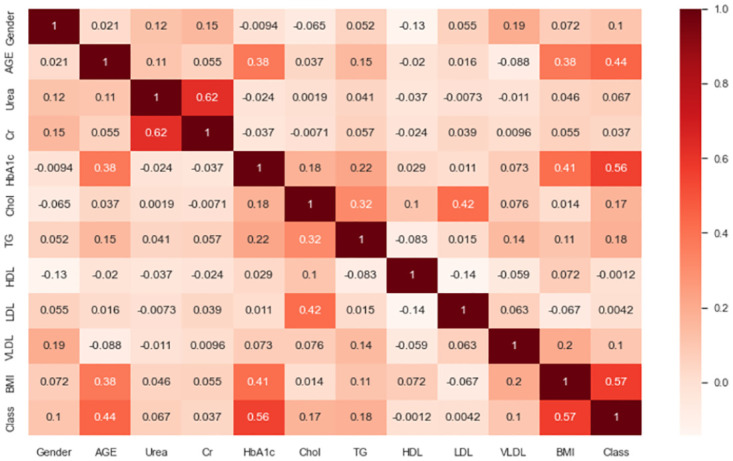
Pearson correlation heatmap for the original extremely imbalanced LMCH data dynamics.

**Fig 5:**
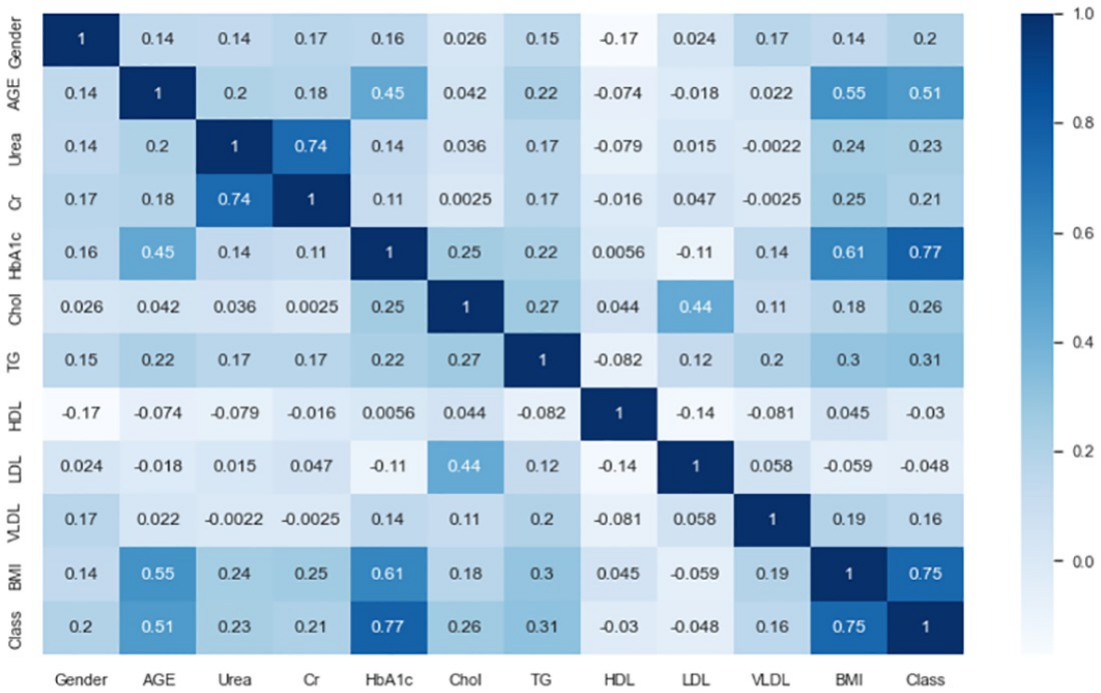
Pearson correlation heatmap for the filtered moderately imbalanced LMCH data dynamics.

Figs 4 and 5 depict the heatmap of the experimental dataset, which provides a visual representation of the correlation matrices showing the correlation between various features. The correlation coefficient ranges from -1 to 1, where -1 indicates a negative correlation and +1 indicates a positive correlation. The figure provides insights into the relationships among the features.

For the original extremely imbalanced LMCH data dynamics, several observations can be made. Firstly, the features *BMI*, *HbA1c*, and *AGE* show the highest correlation coefficients with the *Class* feature, with values of 0.57, 0.56, and 0.44, respectively. Secondly, the features *HDL*, *LDL*, *Cr*, and *Urea* have the lowest correlation coefficients with the *Class* feature. Furthermore, there are feature-feature correlations such as *AGE-HbA1c*, *AGE-BMI*, *Urea-Cr*, *HbA1c-TG*, *HbA1c-BMI*, *Chol-TG*, and *Chol-LDL* with correlation coefficients of 0.32, 0.38, 0.62, 0.22, 0.41, 0.32, and 0.42, respectively. In the filtered moderately imbalanced LMCH data dynamics, similar patterns emerge. The features *HbA1c*, *BMI*, and *AGE* again exhibit the highest correlation coefficients with the *Class* feature, with values of 0.77, 0.75, and 0.51, respectively. The features *HDL* and *LDL* have the lowest correlation coefficients with the *Class* feature, as visually depicted in the correlation matrix. Additionally, there are feature-feature correlations such as *BMI-HbA1c*, *BMI-AGE*, *HbA1c-AGE*, *Cr-Urea*, and *LDL-Chol* with correlation coefficients of 0.61, 0.55, 0.45, 0.74, and 0.44, respectively. From Figs 4 and 5, it is evident that the filtered moderately imbalanced LMCH data dynamics exhibit similar correlation patterns as the original LMCH data dynamics. The heatmap of the filtered data dynamics closely resembles the heatmap of the original data dynamics, indicating that the filtering process has preserved the underlying correlations between features.

### Problems of LMCH dataset to be addressed

The LMCH diabetes dataset exhibits certain issues that may hinder the achievement of optimal classification performance. These problems are listed below:

- Mixture of categorical and numerical data
- Duplicate instances
- Outliers
- Imbalanced class distribution

In our research, we initially discarded the features *ID* and *No_Pation*. After removing these features, the dataset consisted of 11 independent features and one dependent feature.

### Dataset preprocessing

The process of preparing raw data for model development and training is known as data preprocessing. Data preprocessing is a crucial step that enhances the quality of data, enabling models to extract valuable insights. It involves various techniques such as handling missing values, outliers, inconsistent data, normalization, standardization, encoding, feature selection, dimensionality reduction, and more. The data preprocessing techniques used in our research are listed below:

#### Label encoding

The process of transforming a sequence of characters or words into a specific format is known as encoding. Label encoding involves assigning a unique integer number to categorical variables based on their dictionary order. It maps the categories to the range of [0, *n* − 1], where *n* represents the number of categorical values [38].

#### Duplicate instance removal

Duplicate instances refer to multiple copies of the same data instance. When training a machine learning model, having duplicate instances can lead to overfitting and hinder the model’s ability to generalize well. In the case of the original LMCH dataset, which contained 174 duplicate instances, all duplicate instances were discarded except for the first occurrence. As a result, the new dataset consists of 826 unique instances, with 96 (11.62%) representing healthy controls, 40 (4.84%) representing pre-diabetes, and 690 (83.54%) representing diabetes subjects. The created moderately imbalanced LMCH data dynamics do not include any duplicate instances.

#### Z-score outlier detection and handling

Outliers are data points that deviate significantly from the overall pattern of observations in a dataset. They can hurt the training process and lead to less accurate models and inferior outcomes. In this study, the KDE plot in Figs 2 and 3, indicate a Gaussian data distribution. Therefore, the Z-score mechanism was employed for outlier detection and handling, as it is well-suited for this type of data distribution [39]. The Z-score mechanism helps to identify data points that are far from the mean and allows for appropriate treatment of outliers.

#### Standardization

Standardization is a scaling method that centers the values around the mean and scales them to have a unit standard deviation. It is an effective approach when the data distribution of the attributes is Gaussian and when the algorithm assumes the data follows a Gaussian distribution. Standardization is also beneficial when the data contains features with varying scales. Since both the original extremely imbalanced LMCH data dynamics and the filtered moderately imbalanced LMCH data dynamics exhibit a Gaussian-like distribution, as shown in Figs 2 and 3, this paper utilized standardization as a preprocessing step. By applying standardization, the data is transformed to have zero mean and unit variance, which can improve the performance of certain machine learning algorithms and ensure that features are on a similar scale.

### Feature selection

Feature selection is a technique used to reduce the dimensionality of the input space by selecting only the most relevant features and removing irrelevant or noisy ones. In this study, various types of feature selection methods are applied to identify the most informative features for diabetes mellitus prediction. These methods are described below:

#### Filter method

Filtering techniques capture intrinsic properties of traits measured using univariate statistics rather than CV performance [40].

*Information gain*. Information gain (IG) calculates the reduction in entropy due to transforming the dataset [41]. It can be used for feature selection by evaluating the information gain from each variable in the context of the target variable [42]. If features have several potential outcomes, information gain may be biased; this bias can be rectified using gain ratio criterion [43].

#### Wrapper method

The feature selection algorithm in the wrapper method wraps around the predictive model algorithm to select the best features. In this analysis, we utilized two wrapper methods, which are discussed below:

*Backward feature elimination*. Backward elimination improves the model’s performance by starting with all the features and gradually removing the least important one at a time [41]. After running the entire model with all the independent variables included, the irrelevant feature with the highest p-value (above the significance level) is eliminated. The LR algorithm serves as the base model for backward elimination (BFE).

*Sequential feature selection*. Essentially, Sequential feature selection (SFS) algorithms are a subset of wrapper approaches that iteratively add and remove features from the dataset [40]. In SFS, the LR algorithm is used as the base model.

#### Embedded method

In the embedded approach, the feature selection process is integrated into the learning or model-building phases. In this study, the RF-embedded method is utilized.

*RF.*.The RF is a type of bagging algorithm that consists of multiple decision trees. The use of multiple trees helps overcome the limitations of individual trees in terms of precision and overfitting. From a randomly chosen portion of the training dataset, the RF classifier generates several decision trees [44]. The stability and improved performance of RF come from the combination of weakly correlated classifiers and regressions [10].

### ML classifier

We employed several supervised ML classifiers to assess the predictive performance of diabetes mellitus disease. Based on the KDE plot of the original LMCH and filtered LMCH data dynamics, which mostly follow a normal distribution, we selected the following ML classifiers. A brief description of each classifier is provided below:

**SVM.** The SVM attempts to categorize data points in a multidimensional space using the proper hyperplane [45–48]. For implementing multiclass classification, a method called multiclass SVM (MSVM) has been proposed. There are commonly used MSVM classification implementations, such as One-Vs-Rest (OvR) or one-against-all (OAA) and one-against-one (OAO) methods. The OAA-SVM method trains multiple 2-class SVMs to solve an n-class problem. In this study, the OAA method was used to generate a multi-class classifier.

**K-nearest neighbor**. The k-nearest neighbors (KNN) algorithm is a supervised machine learning classifier that uses the proximity of data points to make classifications or predictions [49]. To determine the distances between every new data point and any existing data point, the KNN employs the Euclidean distance function [50]. In this study, we implemented a One-against-all KNN classifier.

**Logistic regression**. In supervised learning, a technique known as logistic regression uses sigmoid function probability evaluation to estimate the relationship between a binary dependent variable and at least one independent variable [45, 51]. In the case of multiclass classification, one approach is the One-vs-All (OvA) logistic regression, also known as the One-vs-Rest (OvR) logistic regression [52]. In this study, we utilized the One-vs-All logistic regression classifier for multiclass classification in the context of diabetes mellitus prediction.

### Result evaluation

To evaluate the performance of several ML classifiers for diabetes mellitus detection, various performance metrics were used. These metrics are described below:

**Confusion matrix**. The confusion matrix, as shown in Table 3, is a performance evaluation mechanism that summarizes the performance of the classification model by calculating true positive (TP), true negative (TN), false positive (FP), and false negative (FN).

**Table 3:** Confusion matrix.

Total Sample	Predicted	
	Predicted Negative	Predicted Positive
**Actual Negative**	TN	FP
**Actual Positive**	FN	TP

- TP: The number of cases that were properly predicted as positive.
- TN: The number of cases that were properly predicted as negative.
- FP: The number of negative cases that were erroneously predicted as positive.
- FN: The number of positive cases that were erroneously predicted as negative.

$$\begin{array}{c} \text{Total}\;\text{Sample}=TP+TN+FP+FN \end{array}$$ (1)

The following performance metrics are calculated from the confusion matrix:

**Accuracy**. Accuracy is defined as the ratio of correctly categorized data instances to all data instances [53]. Mathematically,

$$\begin{array}{c} \text{Accuracy}=\frac{TP+TN}{TP+TN+FP+FN} \end{array}$$ (2)

**Precision**. Precision calculates the correctness of the minority class [54]. Mathematically,

$$\begin{array}{c} \text{Precision}=\frac{TP}{TP+FP} \end{array}$$ (3)

**Recall**. Recall tells us how many of the positive instances out of all the actual positive instances were correctly identified [54, 55]. Mathematically,

$$\begin{array}{c} \text{Recall}=\frac{TP}{TP+FN} \end{array}$$ (4)

**F1-Score**. The F1-score is the harmonic mean of Precision and Recall [53]. It represents the balance between precision and recall, with a higher value indicating a more reliable model. Mathematically,

$$\begin{array}{c} \text{F1-Score}=\frac{2\times \text{Precision}\times \text{Recall}}{\text{Precision}+\text{Recall}} \end{array}$$ (5)

**Cohen Kappa.** In the case of multi-class classification or an imbalanced dataset, assessments like accuracy, precision, or recall may not provide us with accurate performance. Mathematically,

$$\begin{array}{c} Cohen\;Kappa=\frac{P_{o}-P_{e}}{1-P_{e}} \end{array}$$ (6)

Where *P*_*o*_ represents the observed agreement and *P*_*e*_ represents the expected agreement.

**AUC.** The Area Under the Curve (AUC), which serves as a summary of the ROC curve, is a measurement of the classifier’s capacity to distinguish between classes. The performance of the model in separating the positive and negative classes improves with increasing AUC.

**ROC.** The Receiver Operating Characteristic curve, or ROC curve, is an effective tool for comparing the false positive rate (x-axis) and true positive rate (y-axis) for a variety of potential threshold values between 0 and 1.

## Results and discussion

**Fig 6:**
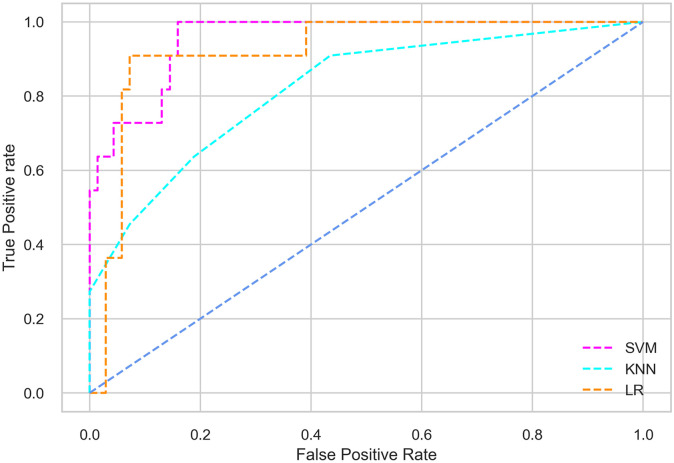
The ROC on MI 70:30 using all features.

**Fig 7:**
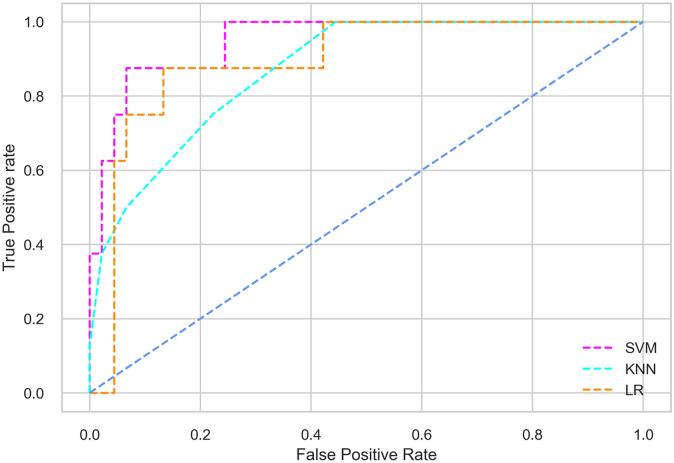
The ROC on MI 80:20 using all features.

**Fig 8:**
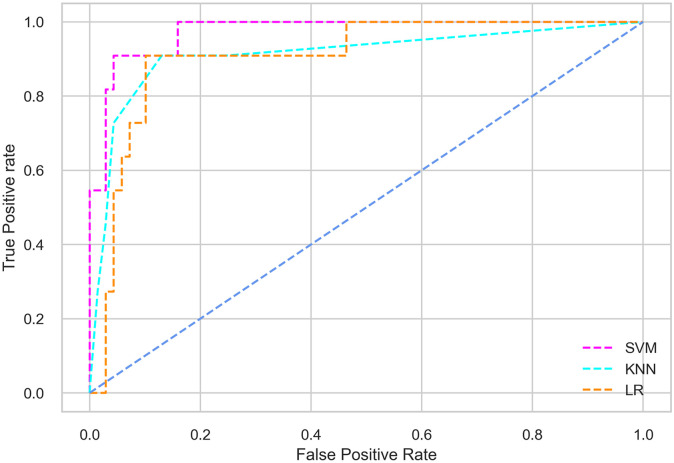
The ROC on MI 70:30 using IG top 4 features.

**Fig 9:**
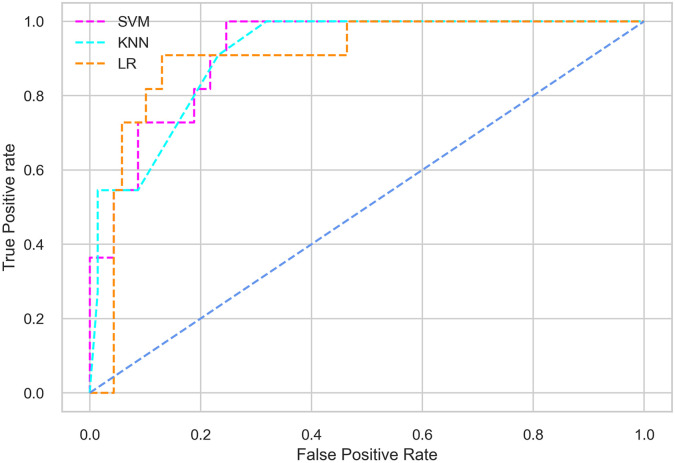
The ROC on MI 70:30 using IG top 5 features.

**Fig 10:**
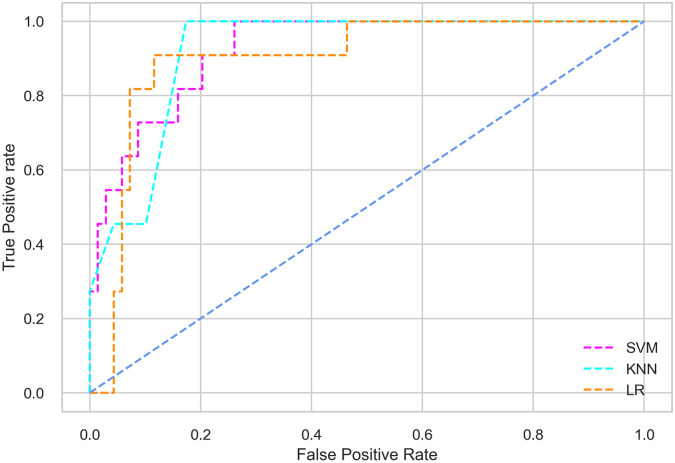
The ROC on MI 7O:30 using IG top 7 features.

**Fig 11:**
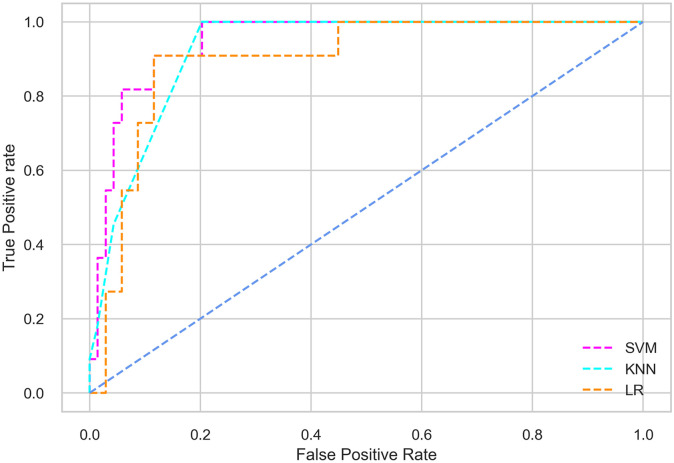
The ROC on MI 70:30 using IG top 9 features.

**Fig 12:**
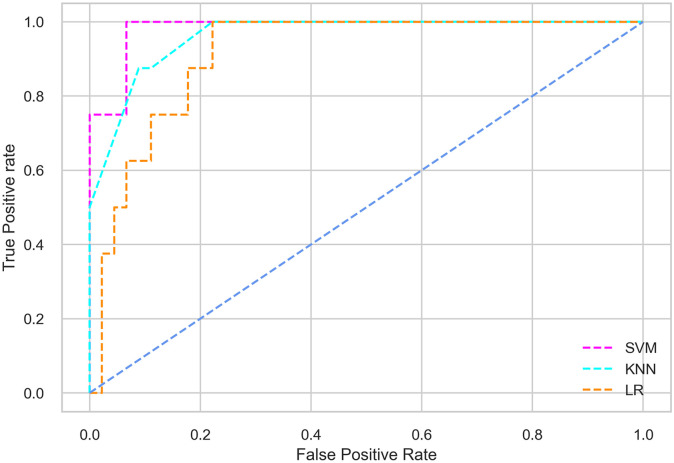
The ROC on MI 80:20 using IG top 4 features.

**Fig 13:**
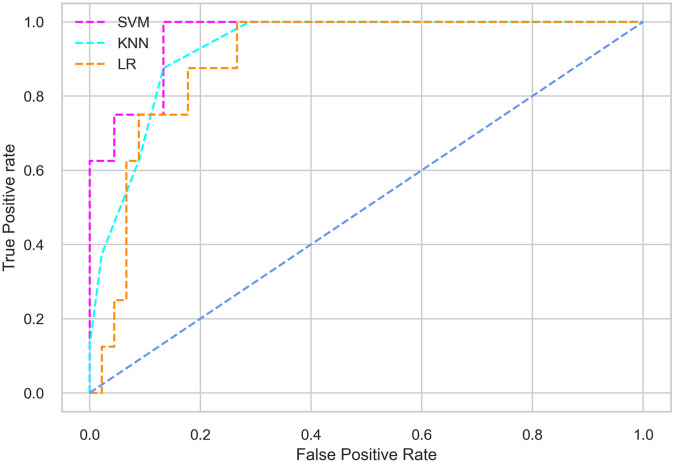
The ROC on MI 80:20 using IG top 5 features.

**Fig 14:**
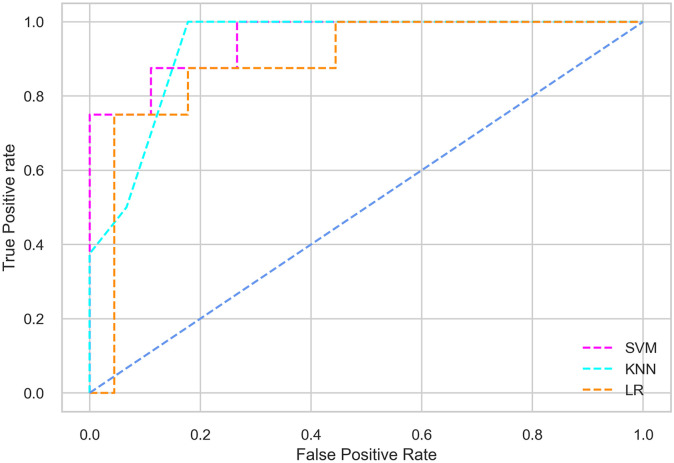
The ROC on MI 80:20 using IG top 7 features.

**Fig 15:**
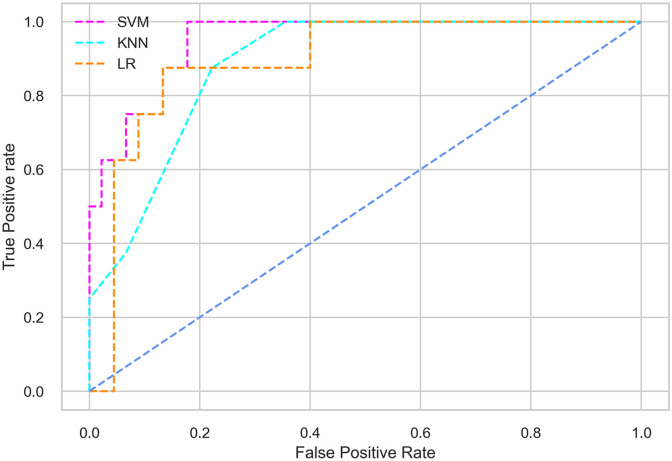
The ROC on MI 80:20 using IG top 9 features.

**Fig 16:**
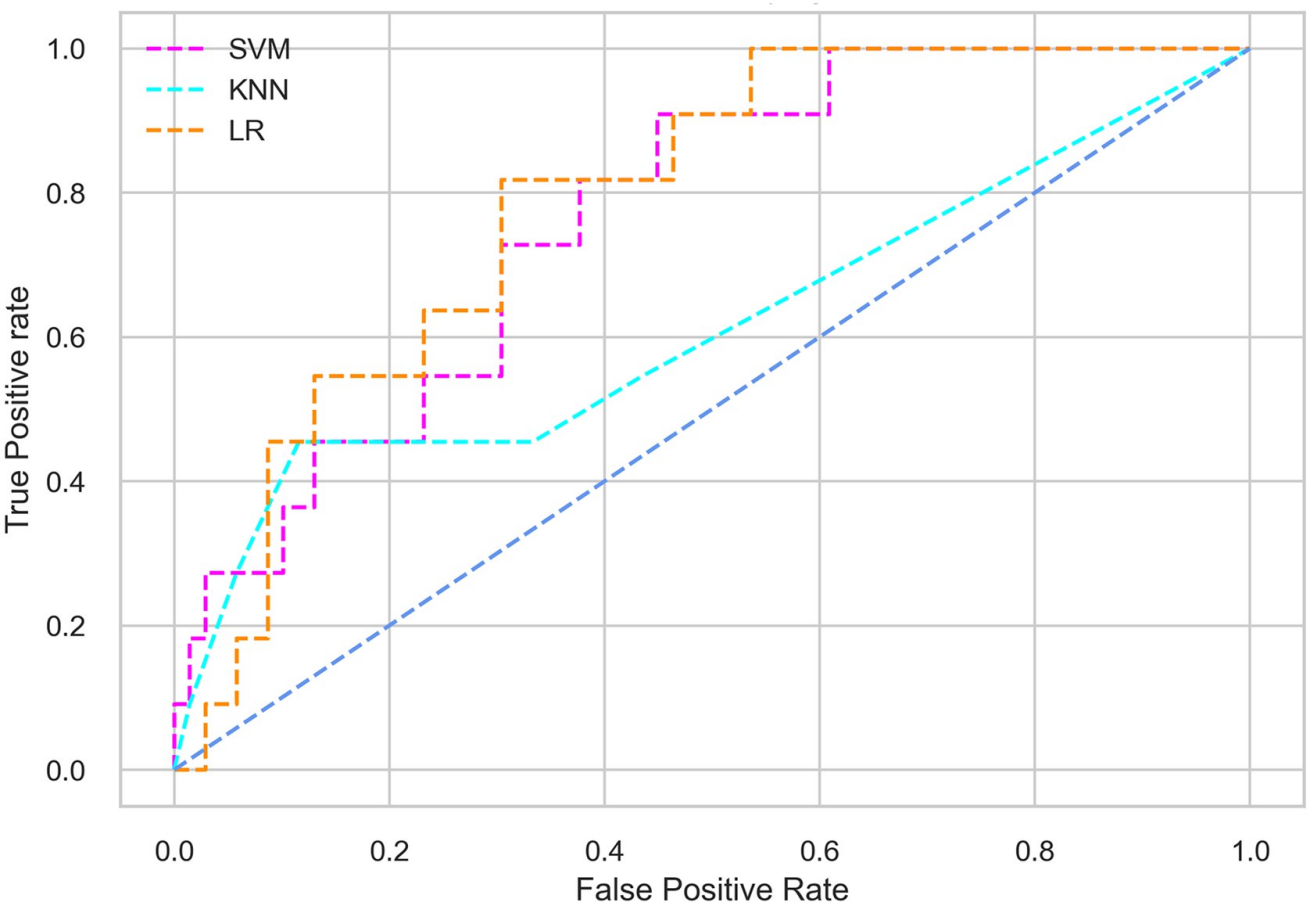
The ROC on MI 70:30 using BFE top 4 features.

**Fig 17:**
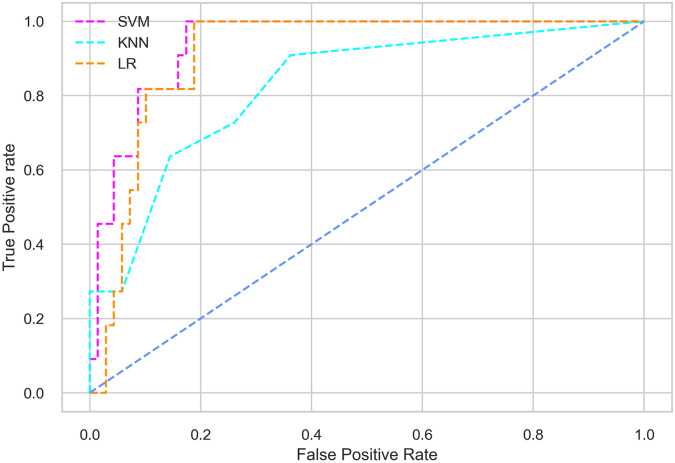
The ROC on MI 70:30 using BFE top 5 features.

**Fig 18:**
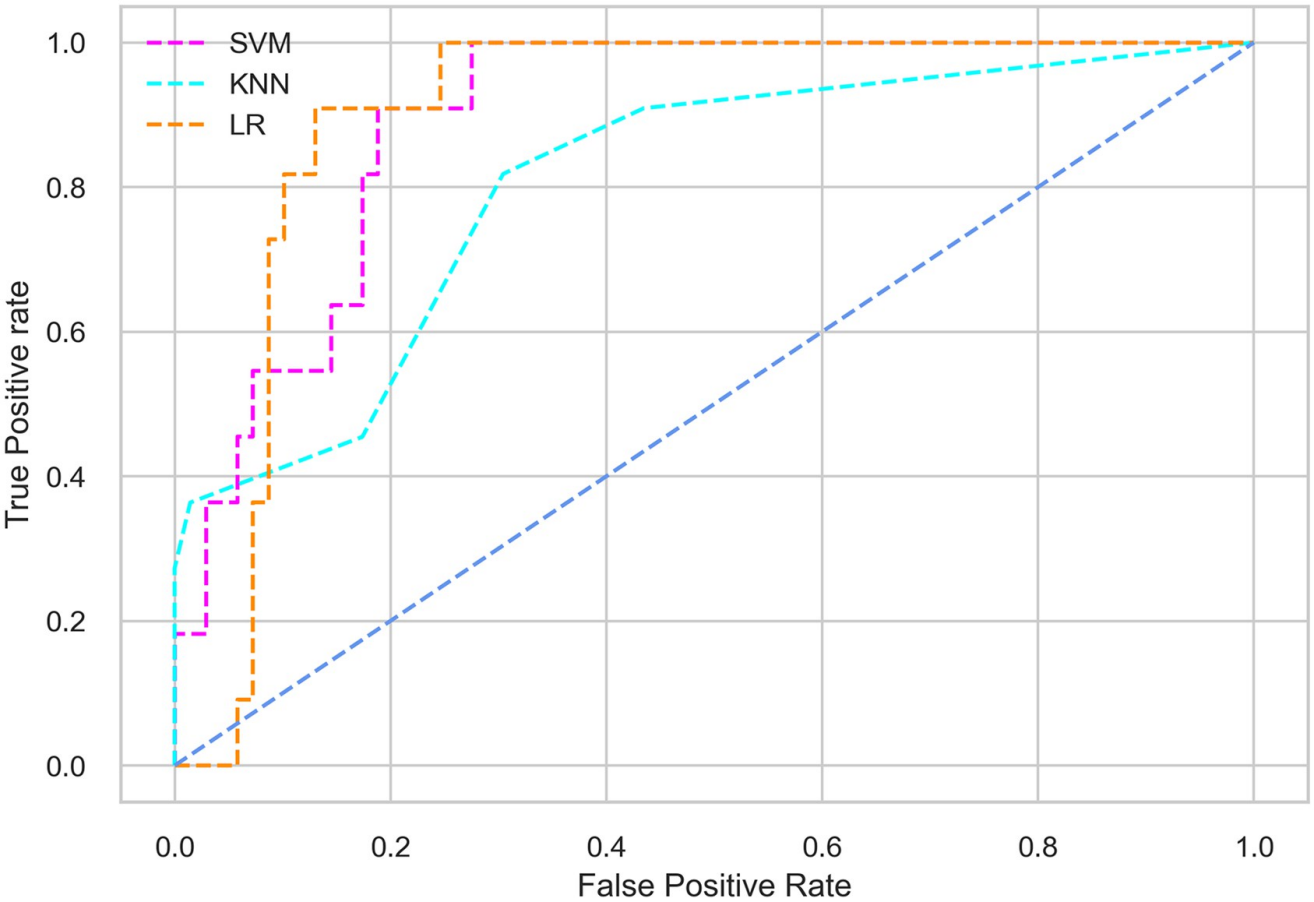
The ROC on MI 70:30 using BFE top 7 features.

**Fig 19:**
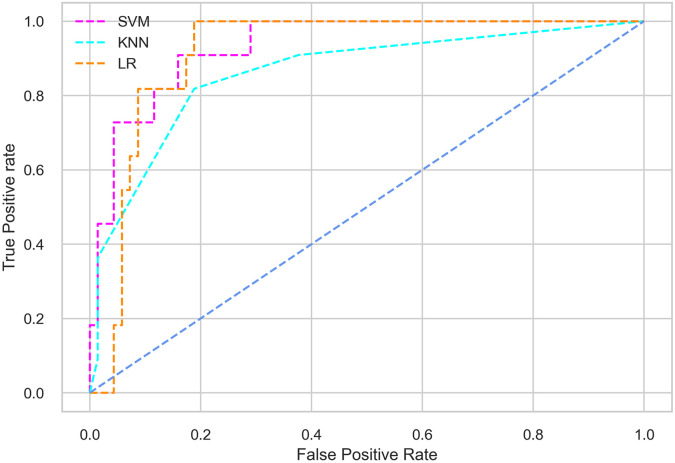
The ROC on MI 70:30 using BFE top 9 features.

**Fig 20:**
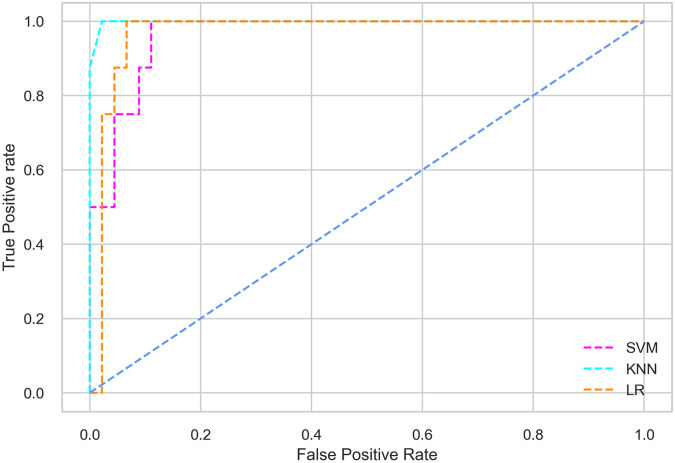
The ROC on MI 80:20 using BFE top 4 features.

**Fig 21:**
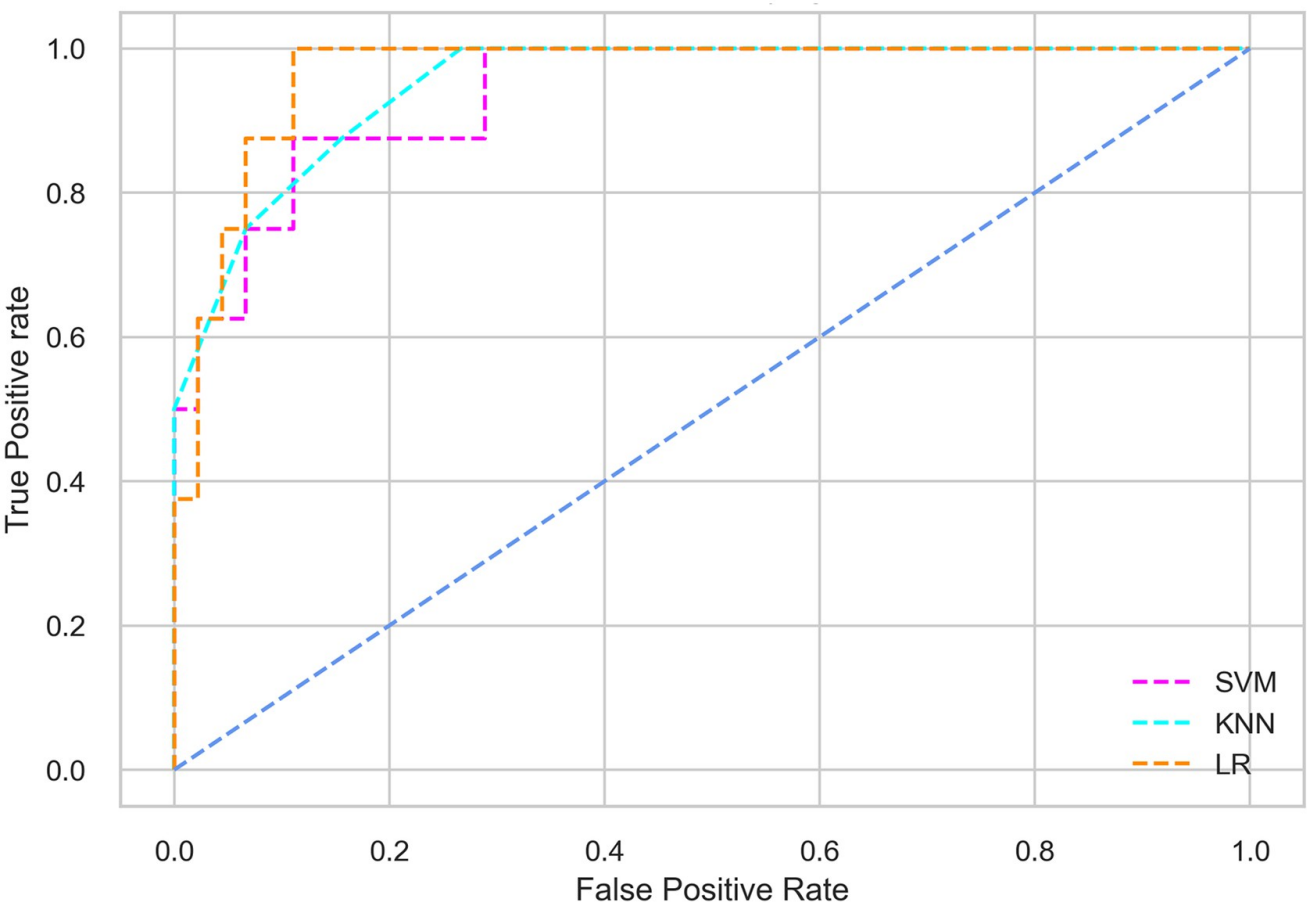
The ROC on MI 80:20 using BFE top 5 features.

**Fig 22:**
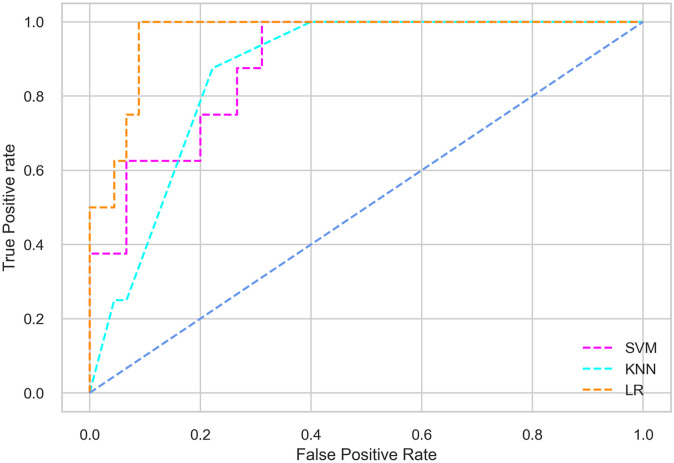
The ROC on MI 80:20 using BFE top 7 features.

**Fig 23:**
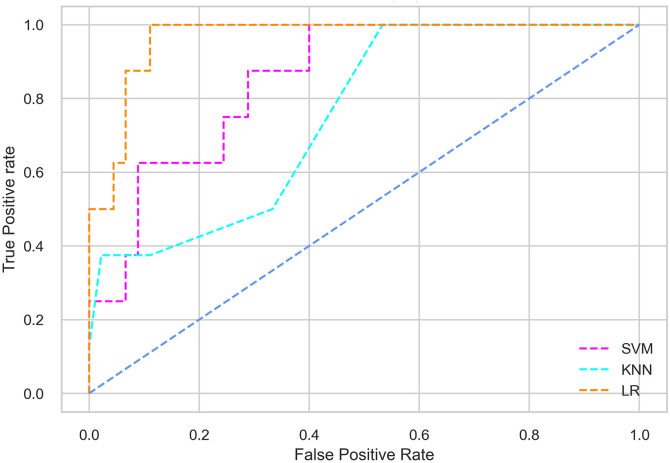
The ROC on MI 80:20 using BFE top 9 features.

**Fig 24:**
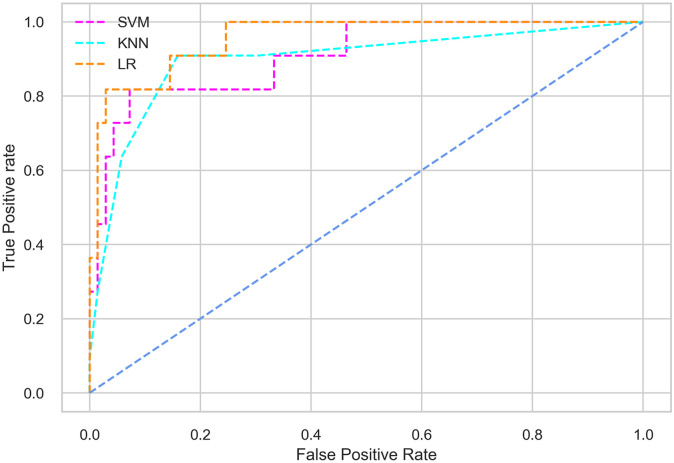
The ROC on MI 70:30 using SFS top 4 features.

**Fig 25:**
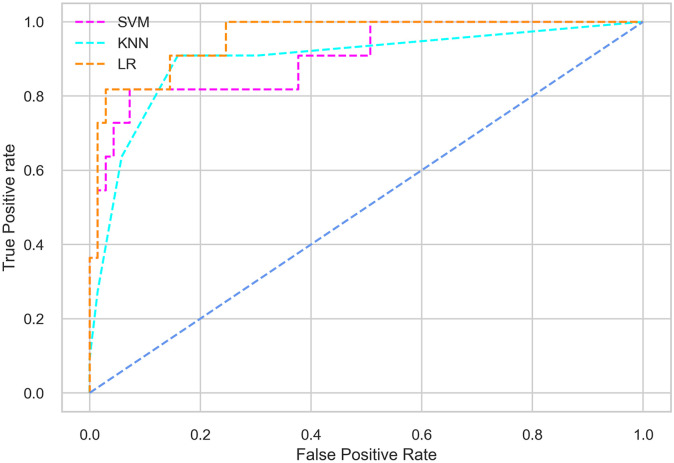
The ROC on MI 70:30 using SFS top 5 features.

**Fig 26:**
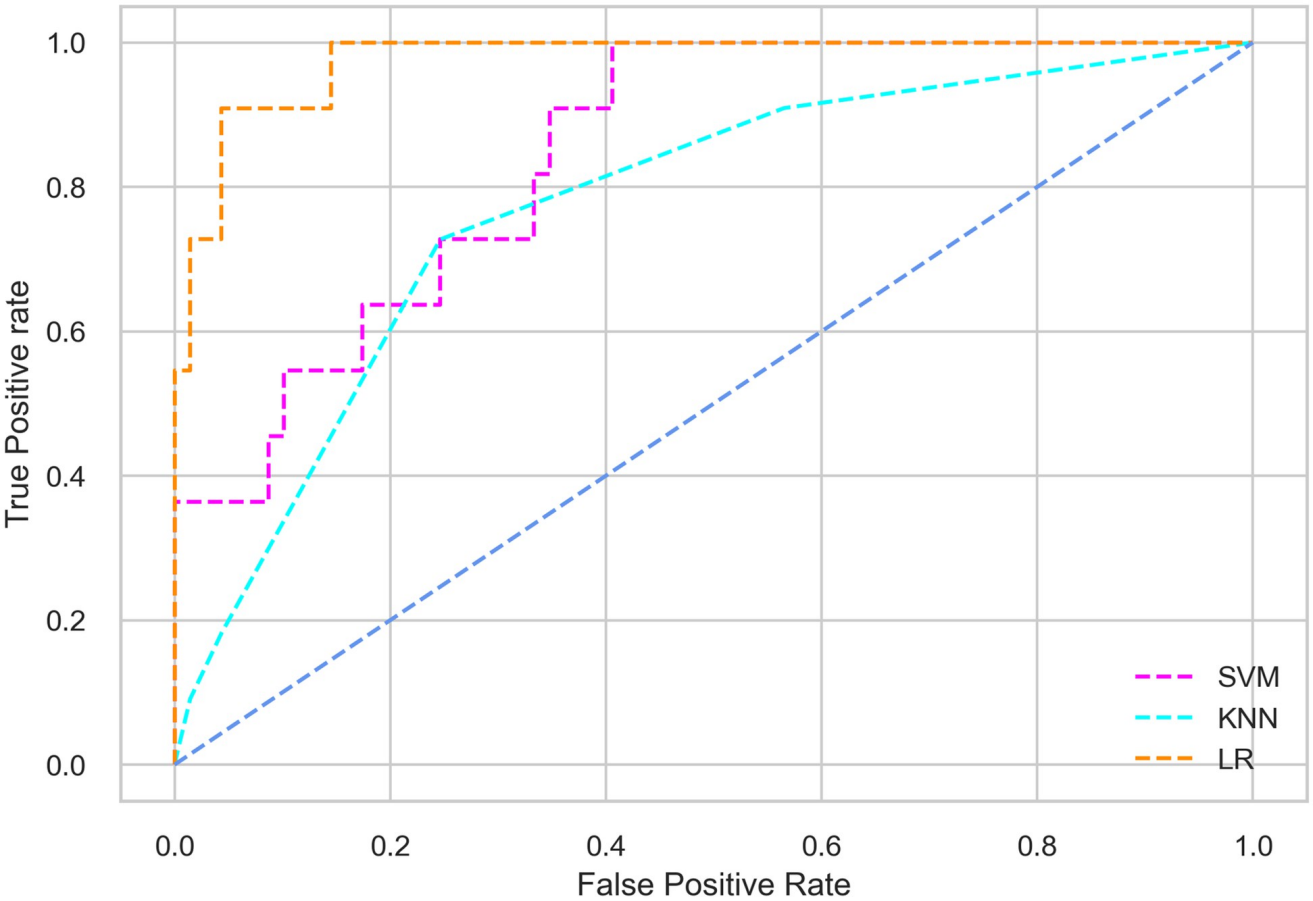
The ROC on MI 70:30 using SFS top 7 features.

Hyperparameter tuning involves the selection of the optimal combination of hyperparameters for a given machine learning algorithm [56, 57]. To ensure the reliability and impartiality of our experiments, we employed a hyperparameter optimization strategy known as Grid Search CV. This method entails systematically exploring a predefined subset of hyperparameters for a given model, assessing each combination through 10-fold CV, and ultimately selecting the best configuration that achieves optimal performance according to a designated evaluation metric. The hyperparameter spaces for SVM, LR, and KNN are listed in Tables 4–6 respectively. The ROC of different machine learning classifiers for numerous feature selection techniques are shows in Figs 6–39. In the case of analysis without hyperparameter optimization and feature selection, we utilized 10-fold CV [58, 59] to avoid bias and ensure a reliable experiment.

**Table 4:** Hyperparameter search space for SVM.

Hyperparameter Name	Hyperparameter Value
C	{x∣1≤x≤20,x∈Z}
Gamma	{0.0001, 0.001, 0.01, 0.1, 1.0}
Kernel	{′*rbf*′, ′*linear*′, ′*poly*′, ′*sigmoid*′}

**Table 5:** Hyperparameter search space for LR.

Hyperparameter Name	Hyperparameter Value
Regularization	*C* = np.logspace(0, 4, 10)
Penalty	{′*l*1′, ′*l*2′}
Solver	{′*liblinear*′, ′*saga*′}

**Table 6:** Hyperparameter Search Space for KNN.

Hyperparameter Name	Hyperparameter Value
N_Neighbors	{x∣1≤x≤20,x∈Z}
Weights	{′*uniform*′, ′*distance*′}
Metric	{′*Minkowski*′, ′*Eclidean*′, ′*Manhattan*′}

### Analysis without utilizing hyperparameter optimization and feature selection techniques

In the analysis phase without applying hyperparameter optimization and feature selection techniques, the maximum performance was achieved by the LR classifier with a 10-fold CV on the moderately imbalanced dataset. On the extremely imbalanced dataset, both the SVM and LR classifiers performed better than KNN. However, these results are much lower than the performance achieved with the application of hyperparameter tuning and feature selection. Table 7 represents the performance of different classifiers on both the moderately imbalanced and extremely imbalanced datasets.

**Table 7:** Analysis before utilizing hyperparameter optimization and feature selection techniques.

Metrics	Moderately Imbalance Dataset			Extremely Imbalance Dataset		
	SVM	LR	KNN	SVM	LR	KNN
Accuracy	0.844	0.893	0.818	0.908	0.909	0.895
Precision	0.841	0.895	0.839	0.921	0.910	0.921
Recall	0.844	0.893	0.818	0.908	0.909	0.895
F1-Score	0.829	0.889	0.816	0.903	0.900	0.896
AUC	0.957	0.975	0.920	0.968	0.976	0.945

### Analysis by utilizing hyperparameter optimization but without applying feature selection based dimensionality reduction

In this stage of the analysis, we utilize the basic preprocessing pipeline. Then, three ML classifiers are trained using the preprocessed dataset. The performance of these classifiers is summarized in Tables 8–11, where *all* means without applying feature selection is indicated in the Top K Selected Feature column and the * symbol indicates without hyper-parameterized result. The optimal hyperparameters for different machine learning settings based on the original extremely imbalanced LMCH dataset are listed in Tables 12 and 13, while the hyperparameters for different machine learning settings based on our proposed the filtered moderately imbalanced LMCH dataset are listed in Tables 14 and 15. To alleviate biases and other negative concerns the 10-fold CV is applied for the reliable experiment.

**Table 8:** Performance comparison of hyper-parameterized classifiers on original LMCH data dynamics using a 70:30 partition with and without feature selection. *Not Used* Indicates without Feature Selection.

FS Method	FS Algorithm	Top K Selected Feature	Model	Accuracy	Precision	Recall	F1-Score	Cohen Kapa	AUC
Not Used	Not Used	all initial features	SVM	0.931	0.963	0.956	0.955	0.804	0.951
Filter*	IG	4		**0.964**	0.968	0.964	0.962	0.835	0.990
		5		0.956	0.965	0.956	0.953	0.804	0.991
		7		0.956	0.968	0.956	0.950	0.806	0.986
		9		0.952	0.955	0.952	0.947	0.770	0.971
Embedded	RF	4		0.943	**0.972**	0.964	0.964	0.844	0.995
		5		0.952	**0.972**	0.964	0.964	0.844	0.989
		7		0.945	0.961	0.956	0.956	0.810	0.987
		9		0.941	0.937	0.935	0.934	0.715	0.959
Wrapper	BFE	4		0.908	0.918	0.907	0.910	0.591	0.935
		5		0.912	0.893	0.907	0.894	0.472	0.932
		7		0.917	0.931	0.935	0.932	0.694	0.955
		9		0.919	0.912	0.919	0.914	0.599	0.961
	SFS	4		0.898	0.854	0.873	0.857	0.640	0.959
		5		0.918	0.951	0.946	0.940	0.836	0.982
		7		0.918	0.951	0.946	0.940	0.836	0.982
		9		0.942	0.972	**0.970**	**0.966**	**0.908**	**0.998**
Not Used	Not Used	all initial features	LR	0.914	0.940	0.940	0.935	0.733	0.973
Filter*	IG	4		**0.944**	**0.955**	**0.944**	**0.938**	0.750	**0.983**
		5		**0.944**	**0.955**	**0.944**	**0.938**	0.750	**0.983**
		7		0.940	0.926	0.940	0.931	0.732	0.978
		9		0.940	0.937	0.940	0.936	0.733	0.977
Embedded	RF	4		0.908	0.935	0.935	0.932	0.719	0.979
		5		0.910	0.925	0.931	0.925	0.709	0.980
		7		0.915	0.922	0.927	0.922	0.688	0.977
		9		0.914	0.922	0.927	0.922	0.688	0.971
Wrapper	BFE	4		0.889	0.882	0.899	0.890	0.520	0.946
		5		0.891	0.902	0.907	0.904	0.567	0.963
		7		0.891	0.913	0.911	0.911	0.603	0.966
		9		0.896	0.905	0.915	0.909	0.625	0.961
	SFS	4		0.892	0.893	0.873	0.863	0.629	0.932
		5		0.903	0.930	0.922	0.908	0.756	0.944
		7		0.903	0.930	0.922	0.908	0.756	0.944
		9		0.920	0.893	0.934	0.912	**0.793**	0.958
Not Used	Not Used	all initial features	KNN	0.926	0.958	0.956	0.955	0.796	0.979
Filter*	IG	4		**0.956**	0.955	0.956	0.953	0.798	0.984
		5		0.952	0.950	0.952	0.949	0.780	0.963
		7		0.948	0.951	0.948	0.949	0.770	0.985
		9		0.948	0.944	0.948	0.945	0.759	0.958
Embedded	RF	4		0.938	0.973	0.972	0.972	0.870	0.994
		5		0.948	0.970	0.968	0.969	0.858	0.992
		7		0.945	0.962	0.960	0.960	0.820	0.990
		9		0.940	0.969	0.964	0.964	0.840	0.956
Wrapper	BFE	4		0.920	0.898	0.895	0.895	0.517	0.961
		5		0.920	0.930	0.935	0.930	0.664	0.972
		7		0.914	0.953	0.956	0.953	0.783	0.976
		9		0.917	0.939	0.944	0.939	0.710	0.954
	SFS	4		0.911	**1.000**	**1.000**	**1.000**	**1.000**	**1.000**
		5		0.921	**1.000**	**1.000**	**1.000**	**1.000**	**1.000**
		7		0.921	**1.000**	**1.000**	**1.000**	**1.000**	**1.000**
		9		0.935	**1.000**	**1.000**	**1.000**	**1.000**	**1.000**

**Table 9:** Performance comparison of hyper-parameterized classifiers on original LMCH data dynamics using an 80:20 partition with and without feature selection. *Not Used* Indicates without Feature Selection.

FS Method	FS Algorithm	Top K Selected Feature	Model	Accuracy	Precision	Recall	F1-Score	Cohen Kapa	AUC
Not Used	Not Used	all initial features	SVM	0.935	0.953	0.940	0.938	0.723	0.956
Filter *	IG	4		0.946	0.964	0.946	0.945	0.761	0.991
		5		0.946	0.964	0.946	0.945	0.761	0.992
		7		0.934	0.960	0.934	0.931	0.714	0.986
		9		0.940	0.946	0.940	0.936	0.703	0.968
Embedded	RF	4		0.950	0.972	0.964	0.966	0.841	0.978
		5		**0.958**	0.953	0.940	0.943	0.741	0.979
		7		0.948	0.943	0.928	0.934	0.696	0.979
		9		0.950	0.938	0.928	0.928	0.682	0.952
Wrapper	BFE	4		0.890	0.826	0.871	0.846	0.581	0.897
		5		0.913	0.848	0.899	0.872	0.663	0.978
		7		0.903	0.915	0.907	0.885	0.688	0.986
		9		0.937	0.944	0.940	0.928	0.802	0.982
	SFS	4		0.898	0.837	0.875	0.852	0.611	0.966
		5		0.918	0.952	0.952	0.950	0.848	0.986
		7		0.918	0.952	0.952	0.950	0.848	0.988
		9		0.942	**0.974**	**0.972**	**0.970**	**0.911**	**0.998**
Not Used	Not Used	all initial features	LR	0.923	**0.942**	0.922	0.918	0.647	0.968
Filter*	IG	4		0.916	0.904	0.916	0.906	0.619	0.979
		5		0.922	0.942	0.922	0.918	0.647	0.980
		7		0.916	0.906	0.916	0.907	0.620	0.975
		9		0.916	0.904	0.916	0.906	0.619	0.974
Embedded	RF	4		0.917	0.902	0.910	0.901	0.601	0.962
		5		0.921	0.900	0.904	0.897	0.583	0.963
		7		0.926	0.900	0.898	0.895	0.558	0.972
		9		0.923	0.900	0.898	0.895	0.558	0.970
Wrapper	BFE	4		0.884	0.822	0.867	0.842	0.564	0.911
		5		0.896	0.829	0.879	0.853	0.592	0.917
		7		0.895	0.826	0.879	0.852	0.577	0.934
		9		**0.927**	0.941	**0.935**	**0.921**	**0.789**	0.977
	SFS	4		0.892	0.873	0.875	0.861	0.602	0.946
		5		0.903	0.917	0.911	0.889	0.693	0.957
		7		0.903	0.917	0.911	0.889	0.693	0.957
		9		0.920	0.934	0.927	0.905	0.758	0.967
Not Used	Not Used	all initial features	KNN	0.938	0.959	0.952	0.951	0.773	0.976
Filter*	IG	4		0.940	0.947	0.940	0.939	0.729	0.986
		5		0.940	0.956	0.940	0.939	0.729	0.959
		7		**0.958**	0.973	0.958	0.962	0.822	0.990
		9		0.940	0.950	0.940	0.937	0.716	0.936
Embedded	RF	4		0.942	0.962	0.952	0.954	0.783	0.993
		5		0.956	0.948	0.940	0.943	0.736	0.987
		7		**0.958**	0.945	0.940	0.940	0.723	0.988
		9		0.952	0.950	0.946	0.944	0.751	0.923
Wrapper	BFE	4		0.904	**1.000**	**1.000**	**1.000**	**1.000**	**1.000**
		5		0.916	**1.000**	**1.000**	**1.000**	**1.000**	**1.000**
		7		0.904	**1.000**	**1.000**	**1.000**	**1.000**	**1.000**
		9		0.929	**1.000**	**1.000**	**1.000**	**1.000**	**1.000**
	SFS	4		0.912	**1.000**	**1.000**	**1.000**	**1.000**	**1.000**
		5		0.921	**1.000**	**1.000**	**1.000**	**1.000**	**1.000**
		7		0.921	**1.000**	**1.000**	**1.000**	**1.000**	**1.000**
		9		0.935	**1.000**	**1.000**	**1.000**	**1.000**	**1.000**

**Table 10:** Performance comparison of hyper-parameterized classifiers on filtered LMCH data dynamics using a 70:30 partition with and without feature selection. *Not Used* Indicates without Feature Selection.

FS Method	FS Algorithm	Top K Selected Feature	Model	Accuracy	Precision	Recall	F1-Score	Cohen Kapa	AUC
Not Used	Not Used	all initial features	SVM	0.892	0.929	0.925	0.926	0.878	0.984
Filter	IG	4		0.946	0.932	0.925	0.927	0.878	0.991
		5		**0.968**	0.919	0.913	0.915	0.858	0.990
		7		0.923	0.928	0.913	0.917	0.859	0.987
		9		0.913	0.894	0.888	0.890	0.817	0.986
Embedded	RF	4		0.946	0.932	0.925	0.927	0.878	0.991
		5		0.968	0.919	0.913	0.915	0.858	0.990
		7		0.930	0.941	0.925	0.930	0.879	0.986
		9		0.908	0.943	0.938	0.939	0.898	0.986
Wrapper	BFE	4		0.750	0.723	0.725	0.718	0.539	0.815
		5		0.912	0.976	0.975	0.975	0.959	0.997
		7		0.901	0.901	0.875	0.883	0.800	0.965
		9		0.880	0.859	0.863	0.861	0.772	0.974
	SFS	4		0.934	0.952	0.950	0.949	0.917	0.996
		5		0.934	0.952	0.950	0.949	0.917	0.996
		7		0.923	**0.988**	**0.988**	**0.987**	**0.979**	**0.999**
		9		0.924	0.976	0.975	0.974	0.958	0.995
Not Used	Not Used	all initial features	LR	0.874	0.935	0.925	0.928	0.879	0.972
Filter	IG	4		0.891	0.938	0.938	0.938	0.897	0.972
		5		0.896	0.930	0.925	0.926	0.877	0.971
		7		0.891	0.939	0.938	0.938	0.897	0.973
		9		0.896	0.939	0.938	0.938	0.897	0.972
Embedded	RF	4		0.891	0.938	0.938	0.938	0.897	0.972
		5		0.896	0.930	0.925	0.926	0.877	0.971
		7		0.880	0.929	0.925	0.926	0.877	0.968
		9		0.880	0.929	0.925	0.926	0.877	0.970
Wrapper	BFE	4		0.690	0.603	0.700	0.648	0.470	0.797
		5		**0.907**	0.929	0.925	0.926	0.877	0.982
		7		0.880	0.906	0.888	0.894	0.819	0.962
		9		0.874	0.898	0.888	0.891	0.816	0.965
	SFS	4		0.874	0.945	0.938	0.934	0.894	0.992
		5		0.874	0.945	0.938	0.934	0.894	0.992
		7		0.863	0.935	0.925	0.919	0.872	**0.995**
		9		0.885	**0.952**	**0.950**	**0.949**	**0.917**	0.980
Not Used	Not Used	all initial features	KNN	0.863	0.826	0.813	0.818	0.696	0.924
Filter	IG	4		0.941	0.865	0.850	0.855	0.759	0.961
		5		**0.946**	0.880	0.863	0.867	0.779	0.970
		7		0.929	0.891	0.875	0.880	0.798	0.975
		9		0.913	0.859	0.850	0.853	0.757	0.975
Embedded	RF	4		0.941	0.865	0.850	0.855	0.759	0.961
		5		**0.946**	0.880	0.863	0.867	0.779	0.970
		7		0.930	0.927	0.925	0.923	0.875	0.973
		9		0.918	0.882	0.875	0.877	0.796	0.976
Wrapper	BFE	4		0.788	0.703	0.700	0.700	0.505	0.800
		5		0.880	0.889	0.875	0.880	0.798	0.952
		7		0.858	0.880	0.875	0.876	0.796	0.962
		9		0.857	0.916	0.913	0.913	0.857	0.970
	SFS	4		0.868	**0.938**	**0.938**	**0.937**	**0.896**	**0.987**
		5		0.868	**0.938**	**0.938**	**0.937**	**0.896**	**0.987**
		7		0.825	0.910	0.913	0.905	0.852	0.963
		9		0.869	0.899	0.900	0.897	0.832	0.975

**Table 11:** Performance comparison of hyper-parameterized classifiers on filtered LMCH data dynamics using an 80:20 partition with and without feature selection. *Not Used* Indicates without Feature Selection.

FS Method	FS Algorithm	Top K Selected Feature	Model	Accuracy	Precision	Recall	F1-Score	Cohen Kapa	AUC
Not Used	Not Used	all initial features	SVM	0.895	0.942	0.943	0.942	0.907	0.986
Filter	IG	4		**0.957**	0.923	0.925	0.923	0.876	0.992
		5		0.943	0.936	0.925	0.928	0.879	0.994
		7		0.943	0.948	0.943	0.945	0.909	0.989
		9		0.914	0.942	0.943	0.942	0.907	0.990
Embedded	RF	4		**0.957**	0.942	0.943	0.942	0.907	0.988
		5		0.943	0.947	0.943	0.944	0.908	0.996
		7		0.943	0.948	0.943	0.945	0.909	0.990
		9		0.919	0.936	0.925	0.928	0.879	0.980
Wrapper	BFE	4		0.938	**1.000**	**1.000**	**1.000**	**1.000**	**1.000**
		5		0.929	0.982	0.981	0.981	0.969	**1.000**
		7		0.900	**1.000**	**1.000**	**1.000**	**1.000**	0.999
		9		0.896	**1.000**	**1.000**	**1.000**	**1.000**	**1.000**
	SFS	4		0.938	**1.000**	**1.000**	**1.000**	**1.000**	**1.000**
		5		0.929	**1.000**	**1.000**	**1.000**	**1.000**	**1.000**
		7		0.900	**1.000**	**1.000**	**1.000**	**1.000**	0.999
		9		0.900	0.942	0.943	0.942	0.907	0.983
Not Used	Not Used	all initial features	LR	0.890	0.914	0.906	0.908	0.848	0.963
Filter	IG	4		**0.919**	0.944	0.943	0.942	0.907	0.987
		5		0.915	0.908	0.906	0.904	0.846	0.976
		7		0.914	0.926	0.925	0.925	0.877	0.960
		9		0.895	0.914	0.906	0.908	0.848	0.961
Embedded	RF	4		0.914	0.926	0.925	0.925	0.877	0.968
		5		0.914	0.942	0.943	0.942	0.907	0.968
		7		0.914	0.926	0.925	0.925	0.877	0.960
		9		0.900	0.926	0.925	0.925	0.877	0.959
Wrapper	BFE	4		0.914	0.982	0.981	**0.981**	**0.969**	**0.998**
		5		0.895	0.937	0.925	0.915	0.873	**0.998**
		7		0.891	0.907	0.906	0.898	0.843	0.994
		9		0.891	0.907	0.906	0.898	0.843	0.994
	SFS	4		0.914	**0.982**	**0.981**	**0.981**	**0.969**	**0.998**
		5		0.895	**0.982**	**0.981**	**0.981**	**0.969**	**0.998**
		7		0.891	0.907	0.906	0.898	0.843	0.994
		9		0.881	0.926	0.925	0.925	0.877	0.956
Not Used	Not Used	all initial features	KNN	0.858	0.888	0.887	0.883	0.811	0.973
Filter	IG	4		**0.944**	0.948	**0.943**	0.945	0.908	0.951
		5		0.924	0.948	**0.943**	0.945	0.908	0.988
		7		0.939	0.928	0.925	0.926	0.878	0.987
		9		0.868	0.924	0.925	0.923	0.876	0.986
Embedded	RF	4		0.939	0.924	0.925	0.923	0.876	0.984
		5		0.924	0.917	0.906	0.909	0.848	0.989
		7		0.939	0.928	0.925	0.926	0.878	0.987
		9		0.901	0.915	0.906	0.908	0.849	0.985
Wrapper	BFE	4		0.914	**0.959**	0.943	**0.946**	**0.910**	**0.997**
		5		0.858	0.924	0.925	0.923	0.876	0.983
		7		0.858	0.884	0.887	0.885	0.814	0.986
		9		0.839	0.884	0.887	0.885	0.814	0.951
	SFS	4		0.914	**0.959**	**0.943**	**0.946**	**0.910**	**0.997**
		5		0.858	0.950	**0.943**	0.945	0.909	0.992
		7		0.858	0.884	0.887	0.885	0.814	0.986
		9		0.872	0.905	0.906	0.904	0.845	0.973

**Table 12:** Hyperparameter optimization with 10-fold grid search CV for the original extremely imbalanced data dynamics using a 70:30 partition with and without feature selection. *Not Used* Indicates without Feature Selection.

FS Method	FS Algorithm	Selected Feature	Model	Hyperparameter Optimization and Values
Not Used	Not Used	all (11) initial features	SVC	{‘C’: 17, ‘gamma’: 0.01, ‘kernel’: ‘rbf’}
Embedded	RF	4		{‘C’: 14, ‘gamma’: 0.1, ‘kernel’: ‘rbf’}
		5		{‘C’: 15, ‘gamma’: 0.1, ‘kernel’: ‘rbf’}
		7		{‘C’: 9, ‘gamma’: 0.1, ‘kernel’: ‘rbf’}
		9		{‘C’: 6, ‘gamma’: 0.1, ‘kernel’: ‘rbf’}
Wrapper	BFE	4		{‘C’: 8, ‘gamma’: 1, ‘kernel’: ‘rbf’}
		5		{‘C’: 11, ‘gamma’: 0.1, ‘kernel’: ‘rbf’}
		7		{‘C’: 20, ‘gamma’: 0.1, ‘kernel’: ‘rbf’}
		9		{‘C’: 18, ‘gamma’: 0.1, ‘kernel’: ‘rbf’}
	SFS	4, 5, 7		{‘C’: 4, ‘gamma’: 1, ‘kernel’: ‘rbf’}
		9		{‘C’: 19, ‘gamma’: 0.1, ‘kernel’: ‘rbf’}
Not Used	Not Used	all (11) initial features	LR	{‘C’: 7.742636826811269, ‘penalty’: ‘l1’, ‘solver’: ‘saga’}
Embedded	RF	4		{‘C’: 2.7825594022071245, ‘penalty’: ‘l1’, ‘solver’: ‘saga’}
		5		{‘C’: 2.7825594022071245, ‘penalty’: ‘l2’, ‘solver’: ‘saga’}
		7		{‘C’: 7.742636826811269, ‘penalty’: ‘l2’, ‘solver’: ‘saga’}
		9		{‘C’: 2.7825594022071245, ‘penalty’: ‘l1’, ‘solver’: ‘saga’}
Wrapper	BFE	4, 5		{‘C’: 1.0, ‘penalty’: ‘l1’, ‘solver’: ‘saga’}
		7		{‘C’: 2.7825594022071245, ‘penalty’: ‘l1’, ‘solver’: ‘saga’}
		9		{‘C’: 1.0, ‘penalty’: ‘l2’, ‘solver’: ‘liblinear’}
	SFS	4, 5		{‘C’: 2.7825594022071245, ‘penalty’: ‘l2’, ‘solver’: ‘saga’}
		7		{‘C’: 1.0, ‘penalty’: ‘l1’, ‘solver’: ‘saga’}
		9		{‘C’: 21.544346900318832, ‘penalty’: ‘l1’, ‘solver’: ‘saga’}
Not Used	Not Used	all (11) initial features	KNN	{‘metric’: ‘manhattan’, ‘n_neighbors’: 15, ‘weights’: ‘distance’}
Embedded	RF	4		{‘metric’: ‘manhattan’, ‘n_neighbors’: 17, ‘weights’: ‘distance’}
		5		{‘metric’: ‘manhattan’, ‘n_neighbors’: 7, ‘weights’: ‘distance’}
		7		{‘metric’: ‘manhattan’, ‘n_neighbors’: 14, ‘weights’: ‘distance’}
		9		{‘metric’: ‘manhattan’, ‘n_neighbors’: 13, ‘weights’: ‘distance’}
Wrapper	BFE	4		{‘metric’: ‘manhattan’, ‘n_neighbors’: 15, ‘weights’: ‘distance’}
		5		{‘metric’: ‘manhattan’, ‘n_neighbors’: 19, ‘weights’: ‘distance’}
		7		{‘metric’: ‘manhattan’, ‘n_neighbors’: 14, ‘weights’: ‘distance’}
		9		{‘metric’: ‘manhattan’, ‘n_neighbors’: 10, ‘weights’: ‘distance’}
	SFS	4, 5		{‘metric’: ‘manhattan’, ‘n_neighbors’: 11, ‘weights’: ‘distance’}
		7		{‘metric’: ‘manhattan’, ‘n_neighbors’: 13, ‘weights’: ‘distance’}
		9		{‘metric’: ‘manhattan’, ‘n_neighbors’: 20, ‘weights’: ‘distance’}

**Table 13:** Hyperparameter optimization with 10-fold grid search CV for the original extremely imbalanced data dynamics using an 80:20 partition with and without feature selection. *Not Used* Indicates without Feature Selection.

FS Method	FS Algorithm	Selected Feature	Model	Hyperparameter Optimization and Values
Not Used	Not Used	all (11) initial features	SVC	{‘C’: 7, ‘gamma’: 1, ‘kernel’: ‘linear’}
Embedded	RF	4		{‘C’: 4, ‘gamma’: 0.1, ‘kernel’: ‘rbf’}
		5		{‘C’: 8, ‘gamma’: 0.1, ‘kernel’: ‘rbf’}
		7		{‘C’: 15, ‘gamma’: 0.1, ‘kernel’: ‘rbf’}
		9		{‘C’: 6, ‘gamma’: 0.1, ‘kernel’: ‘rbf’}
Wrapper	BFE	4		{‘C’: 6, ‘gamma’: 1, ‘kernel’: ‘rbf’}
		5		{‘C’: 11, ‘gamma’: 0.1, ‘kernel’: ‘rbf’}
		7		{‘C’: 16, ‘gamma’: 0.1, ‘kernel’: ‘rbf’}
		9		{‘C’: 19, ‘gamma’: 0.1, ‘kernel’: ‘rbf’}
	SFS	4, 5		{‘C’: 10, ‘gamma’: 1, ‘kernel’: ‘rbf’}
		7		{‘C’: 13, ‘gamma’: 0.01, ‘kernel’: ‘sigmoid’}
		9		{‘C’: 14, ‘gamma’: 0.1, ‘kernel’: ‘rbf’}
Not Used	Not Used	all (11) initial features	LR	{‘C’: 1.0, ‘penalty’: ‘l2’, ‘solver’: ‘saga’}
Embedded	RF	4		{‘C’: 1.0, ‘penalty’: ‘l2’, ‘solver’: ‘saga’}
		5		{‘C’: 3593.813663804626, ‘penalty’: ‘l1’, ‘solver’: ‘saga’}
		7, 9		{‘C’: 2.7825594022071245, ‘penalty’: ‘l1’, ‘solver’: ‘saga’}
Wrapper	BFE	4		{‘C’: 1.0, ‘penalty’: ‘l2’, ‘solver’: ‘saga’}
		5, 7		{‘C’: 1.0, ‘penalty’: ‘l1’, ‘solver’: ‘saga’}
		9		{‘C’: 7.742636826811269, ‘penalty’: ‘l2’, ‘solver’: ‘liblinear’}
	SFS	4, 5		{‘C’: 2.7825594022071245, ‘penalty’: ‘l2’, ‘solver’: ‘liblinear’}
		7		{‘C’: 2.7825594022071245, ‘penalty’: ‘l1’, ‘solver’: ‘liblinear’}
		9		{‘C’: 7.742636826811269, ‘penalty’: ‘l1’, ‘solver’: ‘saga’}
Not Used	Not Used	all (11) initial features	KNN	{‘metric’: ‘manhattan’, ‘n_neighbors’: 6, ‘weights’: ‘distance’}
Embedded	RF	4		{‘metric’: ‘manhattan’, ‘n_neighbors’: 20, ‘weights’: ‘distance’}
		5		{‘metric’: ‘manhattan’, ‘n_neighbors’: 8, ‘weights’: ‘distance’}
		7		{‘metric’: ‘manhattan’, ‘n_neighbors’: 14, ‘weights’: ‘distance’}
		9		{‘metric’: ‘manhattan’, ‘n_neighbors’: 16, ‘weights’: ‘distance’}
Wrapper	BFE	4		{‘metric’: ‘manhattan’, ‘n_neighbors’: 19, ‘weights’: ‘distance’}
		5, 7		{‘metric’: ‘manhattan’, ‘n_neighbors’: 13, ‘weights’: ‘distance’}
		9		{‘metric’: ‘manhattan’, ‘n_neighbors’: 10, ‘weights’: ‘distance’}
	SFS	4, 5		{‘metric’: ‘manhattan’, ‘n_neighbors’: 11, ‘weights’: ‘distance’}
		7		{‘metric’: ‘manhattan’, ‘n_neighbors’: 9, ‘weights’: ‘distance’}
		9		{‘metric’: ‘manhattan’, ‘n_neighbors’: 18, ‘weights’: ‘distance’}

**Table 14:** Hyperparameter optimization using 10-fold grid search CV for the filtered LMCH data dynamics using a 70:30 partition with feature selection.

FS Method	FS Algorithm	Selected Feature	Hyperparameter Optimization and Values
SVM	RF	4	{‘C’: 15, ‘gamma’: 0.1, ‘kernel’: ‘rbf’}
	RF	5	{‘C’: 6, ‘gamma’: 0.1, ‘kernel’: ‘rbf’}
	RF	7	{‘C’: 7, ‘gamma’: 0.1, ‘kernel’: ‘rbf’}
	RF	9	{‘C’: 9, ‘gamma’: 1, ‘kernel’: ‘linear’}
	BFE	4	{‘C’: 1, ‘gamma’: 1, ‘kernel’: ‘rbf’}
	BFE	5	{‘C’: 14, ‘gamma’: 1, ‘kernel’: ‘linear’}
	BFE	7	{‘C’: 18, ‘gamma’: 0.1, ‘kernel’: ‘rbf’}
	BFE	9	{‘C’: 6, ‘gamma’: 1, ‘kernel’: ‘linear’}
	SFS	4,5,7	{‘C’: 10, ‘gamma’: 1, ‘kernel’: ‘linear’}
	SFS	9	{‘C’: 6, ‘gamma’: 1, ‘kernel’: ‘linear’}
LR	RF	4	{‘C’: 2.7825594022071245, ‘penalty’: ‘l2’, ‘solver’: ‘saga’}
	RF	5	{‘C’: 7.742636826811269, ‘penalty’: ‘l2’, ‘solver’: ‘saga’}
	RF	7,9	{‘C’: 1.0, ‘penalty’: ‘l1’, ‘solver’: ‘saga’}
	BFE	4	{‘C’: 1.0, ‘penalty’: ‘l1’, ‘solver’: ‘liblinear’}
	BFE	5,9	{‘C’: 2.7825594022071245, ‘penalty’: ‘l1’, ‘solver’: ‘saga’}
	BFE	7	{‘C’: 21.544346900318832, ‘penalty’: ‘l1’, ‘solver’: ‘saga’}
	SFS	4,5,7,9	{‘C’: 1.0, ‘penalty’: ‘l1’, ‘solver’: ‘saga’}
KNN	RF	4	{‘metric’: ‘manhattan’, ‘n_neighbors’: 3, ‘weights’: ‘distance’}
	RF	5	{‘metric’: ‘manhattan’, ‘n_neighbors’: 13, ‘weights’: ‘distance’}
	RF	7	{‘metric’: ‘manhattan’, ‘n_neighbors’: 10, ‘weights’: ‘distance’}
	RF	9	{‘metric’: ‘manhattan’, ‘n_neighbors’: 6, ‘weights’: ‘distance’}
	BFE	4, 7	{‘metric’: ‘manhattan’, ‘n_neighbors’: 18, ‘weights’: ‘distance’}
	BFE	5	{‘metric’: ‘manhattan’, ‘n_neighbors’: 6, ‘weights’: ‘distance’}
	BFE	9	{‘metric’: ‘manhattan’, ‘n_neighbors’: 20, ‘weights’: ‘distance’}
	SFS	4,5	{‘metric’: ‘manhattan’, ‘n_neighbors’: 20, ‘weights’: ‘distance’}
	SFS	7	{‘metric’: ‘manhattan’, ‘n_neighbors’: 15, ‘weights’: ‘distance’}
	SFS	9	{‘metric’: ‘manhattan’, ‘n_neighbors’: 5, ‘weights’: ‘distance’}

**Table 15:** Hyperparameter optimization using 10-fold grid search CV for the filtered LMCH data dynamics with 80:20 partition with feature selection.

FS Method	FS Algorithm	Selected Feature	Hyperparameter Optimization and Values
SVC	RF	4	{‘C’: 7, ‘gamma’: 1, ‘kernel’: ‘linear’}
	RF	5	{‘C’: 19, ‘gamma’: 0.1, ‘kernel’: ‘rbf’}
	RF	7,9	{‘C’: 11, ‘gamma’: 0.1, ‘kernel’: ‘rbf’}
	BFE	4	{‘C’: 9, ‘gamma’: 1, ‘kernel’: ‘linear’}
	BFE	5,7	{‘C’: 7, ‘gamma’: 1, ‘kernel’: ‘linear’}
	BFE	9	{‘C’: 14, ‘gamma’: 1, ‘kernel’: ‘linear’}
	SFS	4	{‘C’: 9, ‘gamma’: 1, ‘kernel’: ‘linear’}
	SFS	5,7	{‘C’: 7, ‘gamma’: 1, ‘kernel’: ‘linear’}
	SFS	9	{‘C’: 6, ‘gamma’: 1, ‘kernel’: ‘linear’}
LR	RF	4	{‘C’: 7.742636826811269, ‘penalty’: ‘l1’, ‘solver’: ‘saga’}
	RF	5,7,9	{‘C’: 2.7825594022071245, ‘penalty’: ‘l1’, ‘solver’: ‘saga’}
	BFE	4,5,7,9	{‘C’: 1.0, ‘penalty’: ‘l1’, ‘solver’: ‘saga’}
	SFS	4,5,7	{‘C’: 1.0, ‘penalty’: ‘l1’, ‘solver’: ‘saga’}
	SFS	9	{‘C’: 2.7825594022071245, ‘penalty’: ‘l1’, ‘solver’: ‘saga’}
KNN	RF	4	{‘metric’: ‘manhattan’, ‘n_neighbors’: 7, ‘weights’: ‘distance’}
	RF	5	{‘metric’: ‘manhattan’, ‘n_neighbors’: 7, ‘weights’: ‘uniform’}
	RF	7,9	{‘metric’: ‘manhattan’, ‘n_neighbors’: 6, ‘weights’: ‘distance’}
	BFE	4	{‘metric’: ‘manhattan’, ‘n_neighbors’: 17, ‘weights’: ‘distance’}
	BFE	5	{‘metric’: ‘manhattan’, ‘n_neighbors’: 14, ‘weights’: ‘distance’}
	BFE	7	{‘metric’: ‘manhattan’, ‘n_neighbors’: 19, ‘weights’: ‘distance’}
	BFE	9	{‘metric’: ‘manhattan’, ‘n_neighbors’: 20, ‘weights’: ‘distance’}
	SFS	4	{‘metric’: ‘manhattan’, ‘n_neighbors’: 17, ‘weights’: ‘distance’}
	SFS	5	{‘metric’: ‘manhattan’, ‘n_neighbors’: 14, ‘weights’: ‘distance’}
	SFS	7	{‘metric’: ‘manhattan’, ‘n_neighbors’: 19, ‘weights’: ‘distance’}
	SFS	9	{‘metric’: ‘manhattan’, ‘n_neighbors’: 9, ‘weights’: ‘distance’}

**Fig 27:**
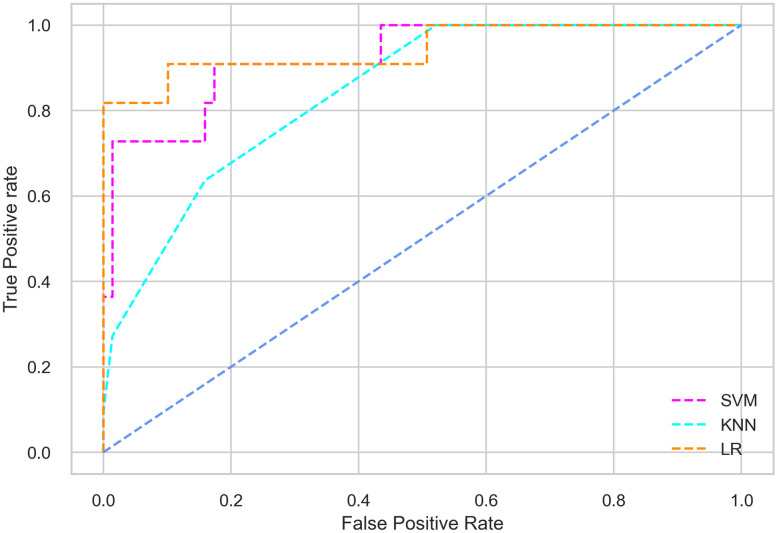
The ROC on MI 70:30 using SFS top 9 features.

**Fig 28:**
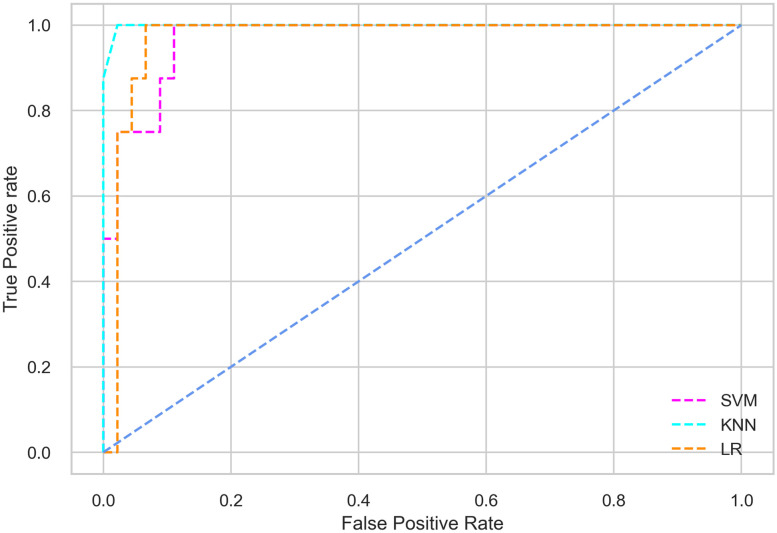
The ROC on MI 80:20 using SFS top 4 features.

**Fig 29:**
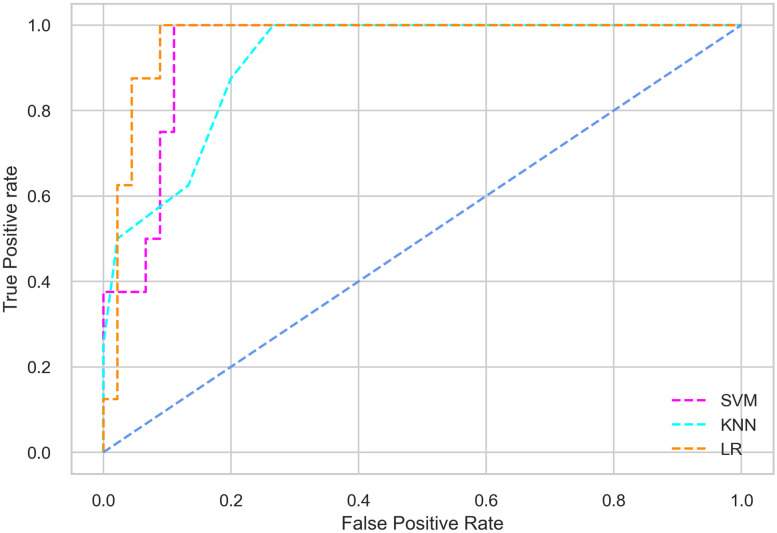
The ROC on MI 80:20 using SFS top 5 features.

**Fig 30:**
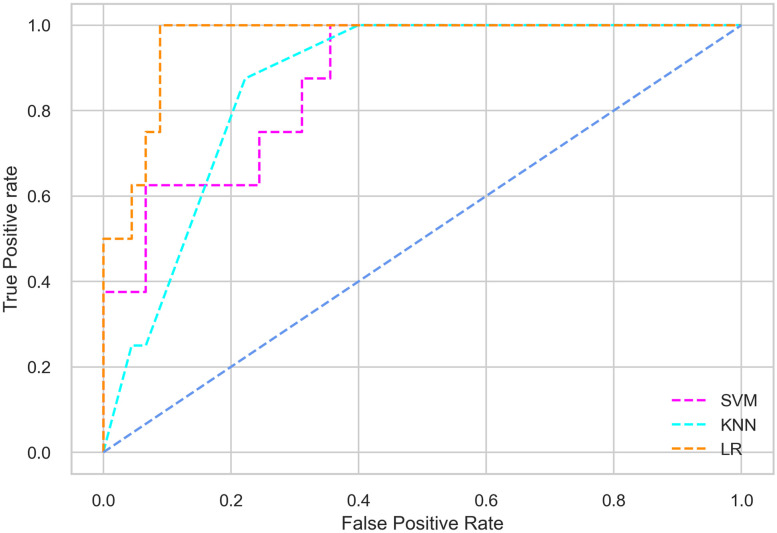
The ROC on MI 80:20 using SFS top 7 features.

**Fig 31:**
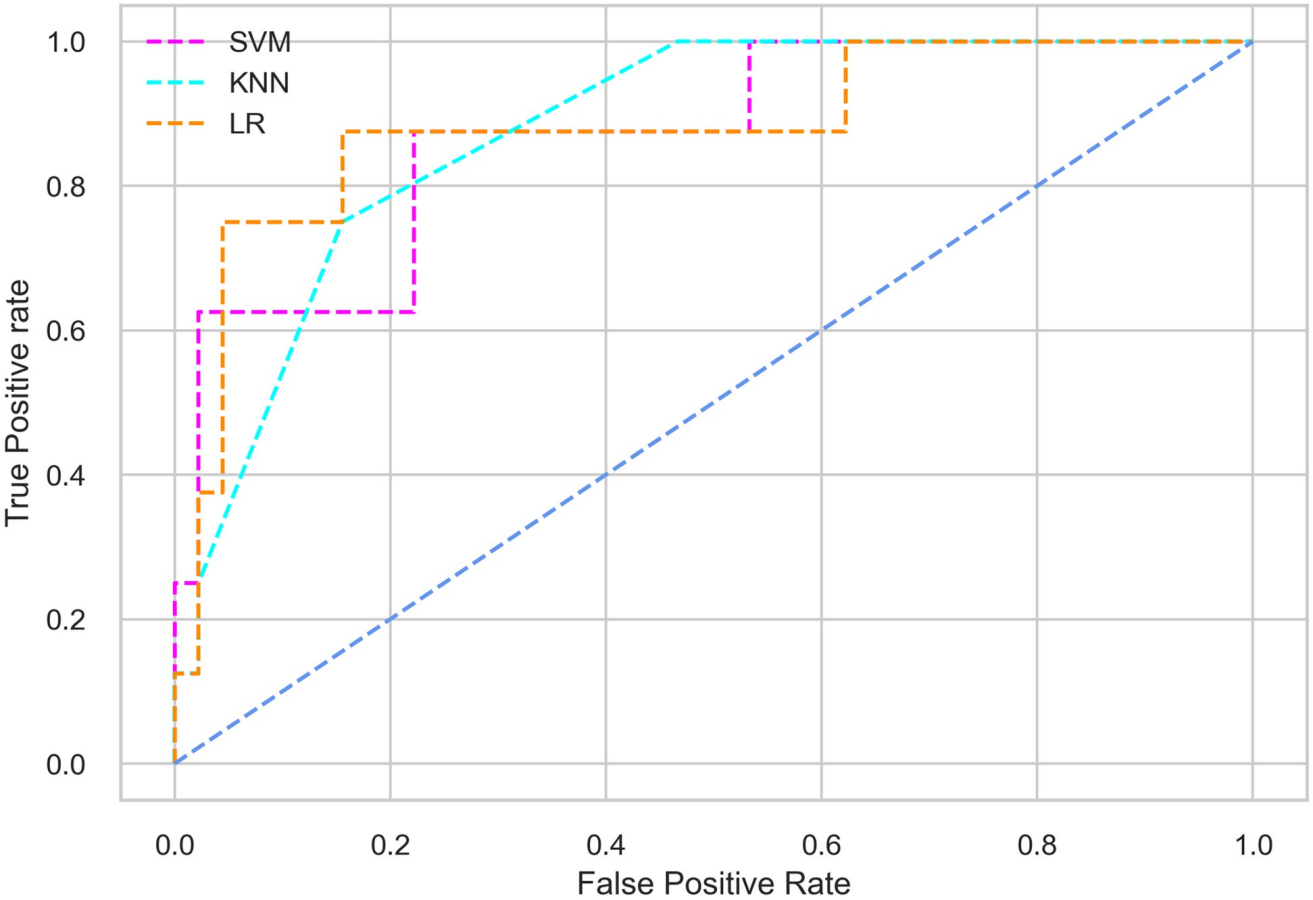
The ROC on MI 80:20 using SFS top 9 features.

**Fig 32:**
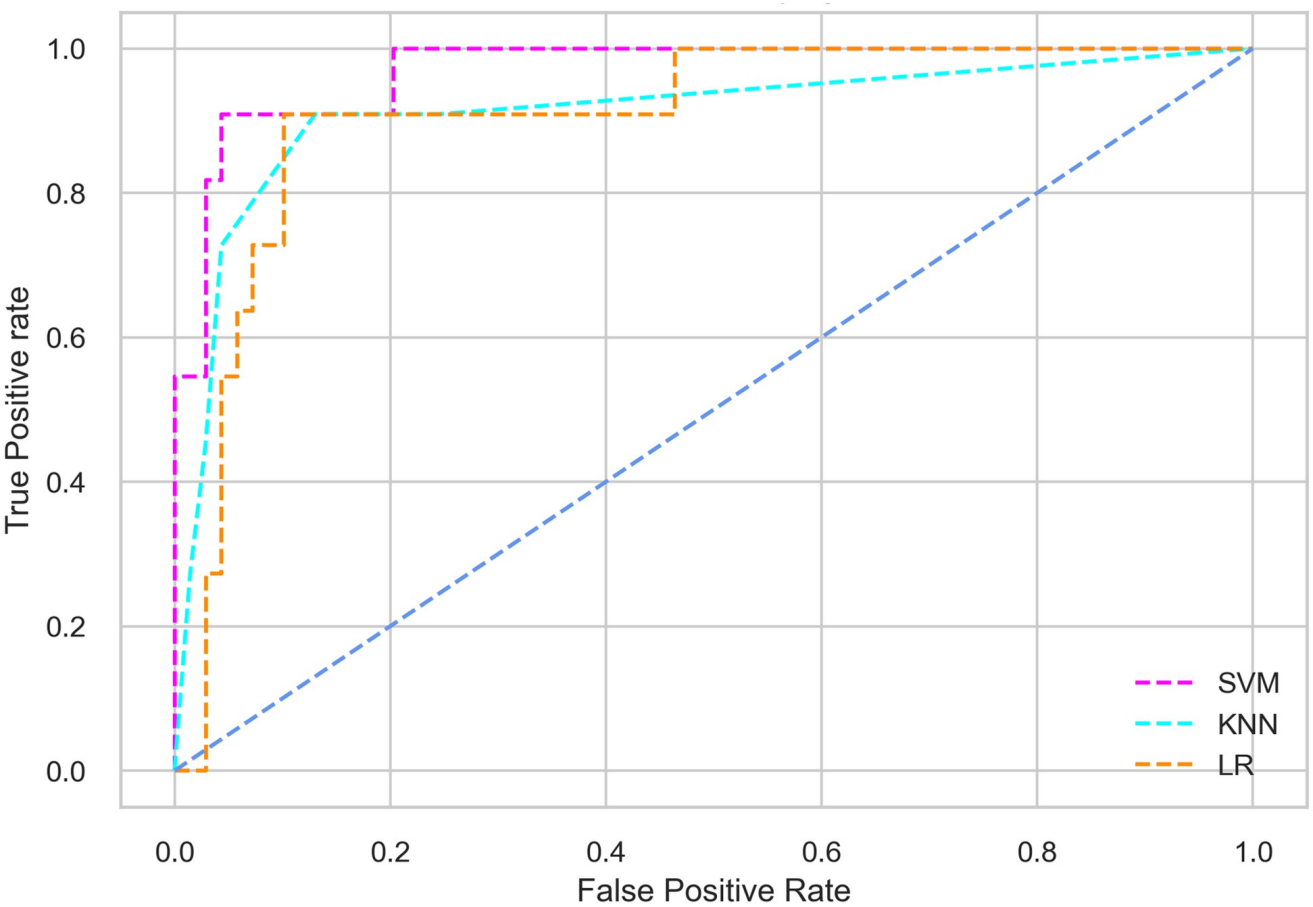
The ROC on MI 70:30 using RF top 4 features.

**Fig 33:**
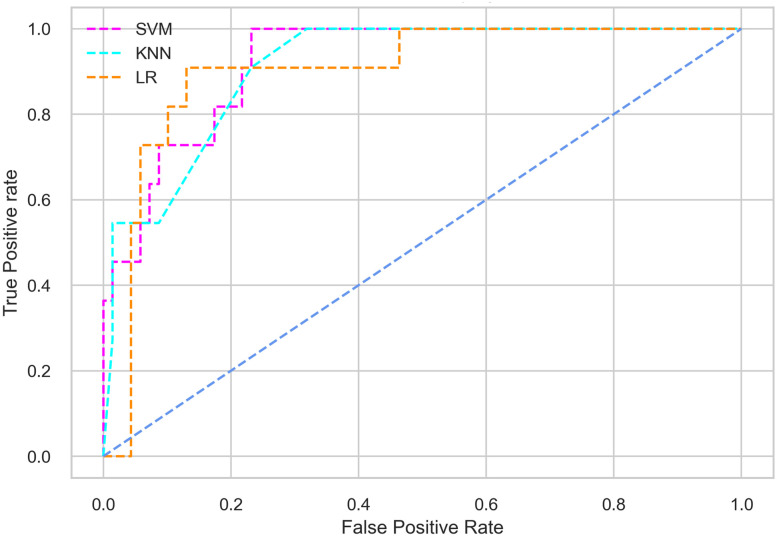
The ROC on MI 70:30 using RF top 5 features.

**Fig 34:**
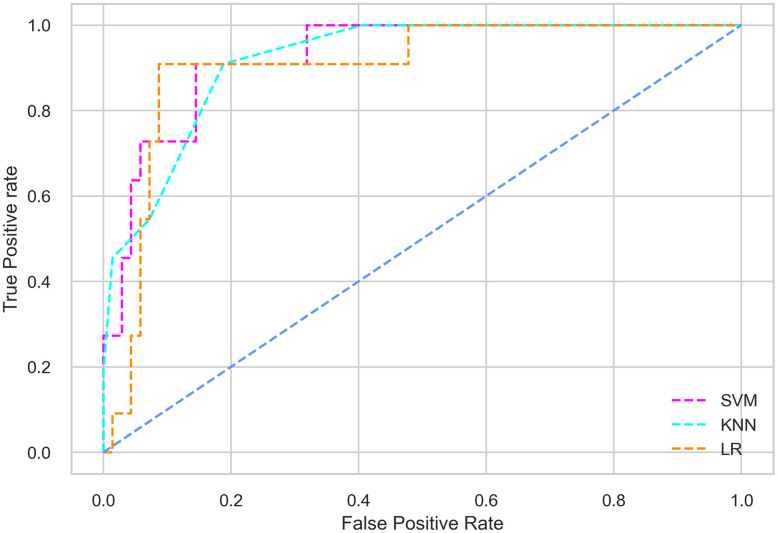
The ROC on MI 70:30 using RF top 7 features.

**Fig 35:**
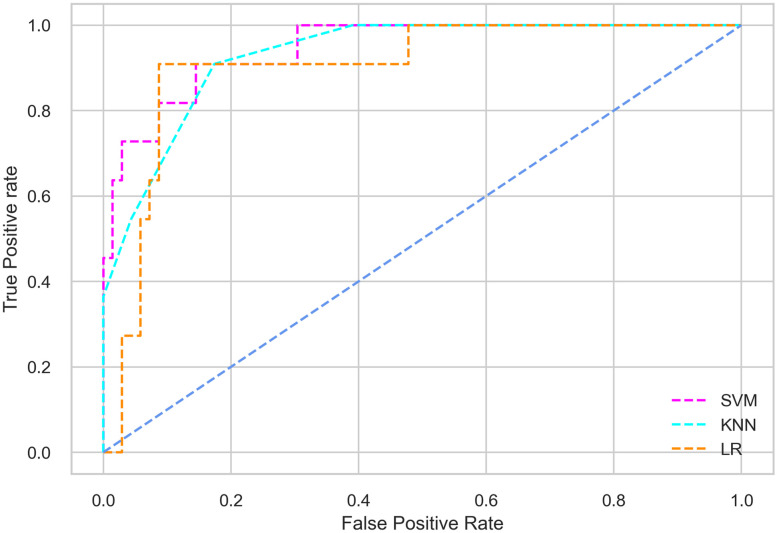
The ROC on MI 70:30 using RF top 9 features.

**Fig 36:**
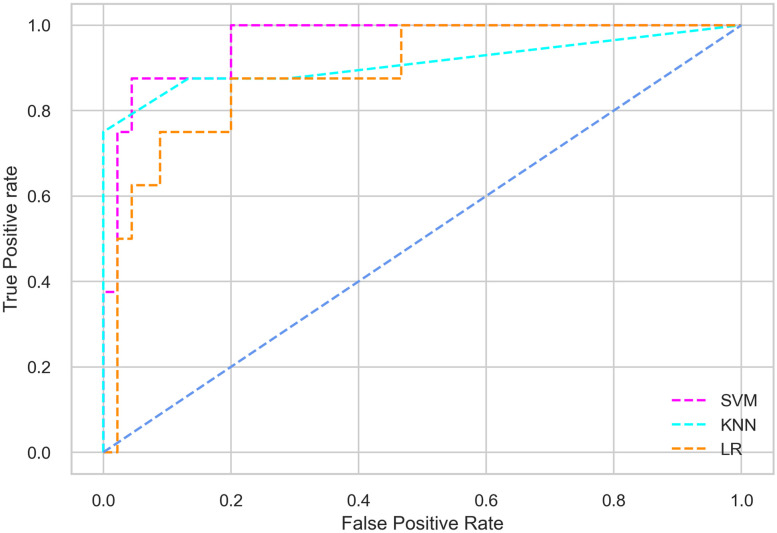
The ROC on MI 80:20 using RF top 4 features.

**Fig 37:**
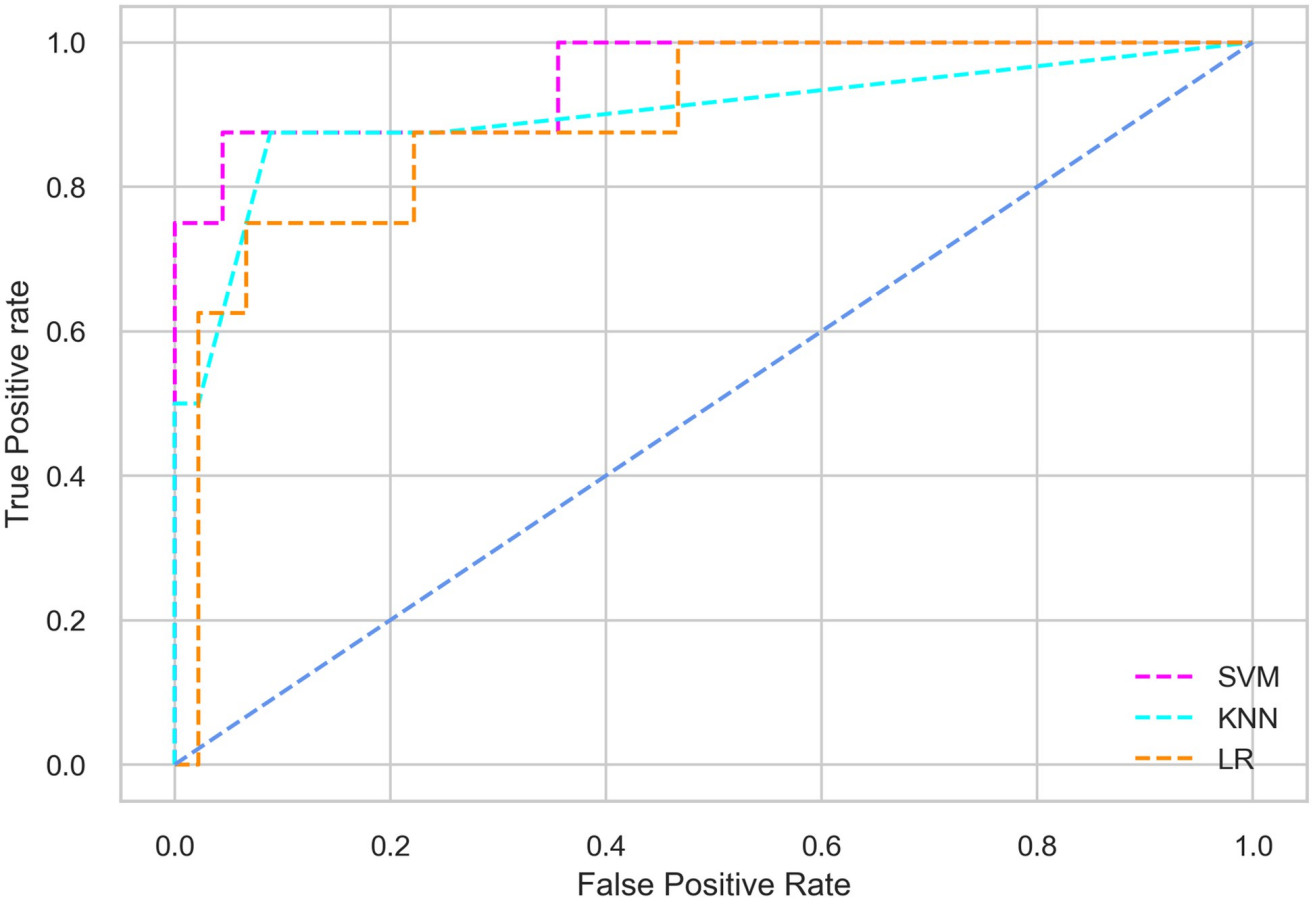
The ROC on MI 80:20 using RF top 5 features.

**Fig 38:**
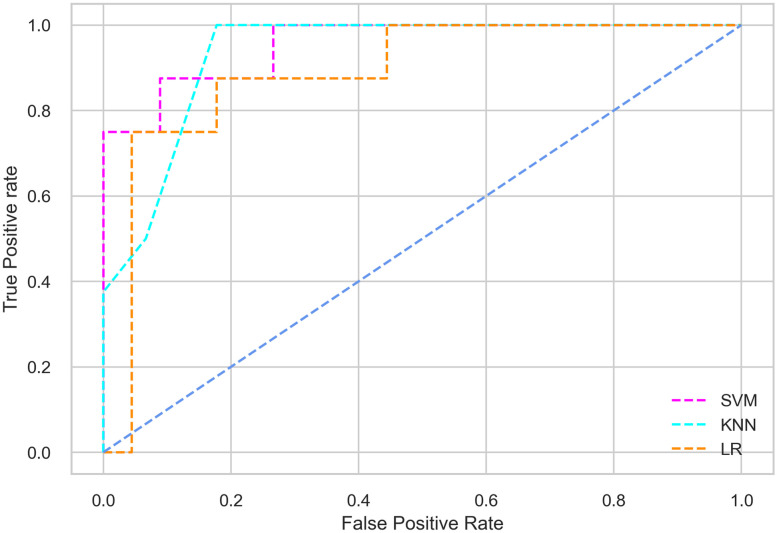
The ROC on MI 80:20 using RF top 7 features.

**Fig 39:**
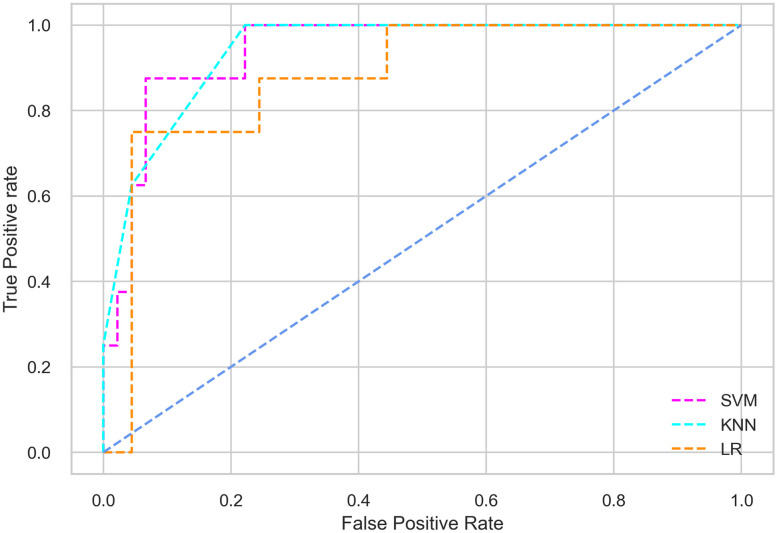
The ROC on MI 80:20 using RF top 9 features.

#### Performance on the original LMCH data dynamics with 70:30 partition

Among the three classifiers, the SVM classifier performs better than the others, except for the AUC metric of 0.951, which is slightly lower than the AUC of 0.979 achieved by the KNN classifier. In general, all the classifiers exhibit similar performance, except for the LR classifier in terms of the Cohen Kappa metric.

#### Performance on the original LMCH data dynamics with 80:20 partition

The KNN classifier achieves the highest scores among the three classifiers for all performance metrics, with an accuracy of 0.938, precision of 0.959, recall of 0.952, F1-score of 0.951, Cohen kappa of 0.773, and AUC of 0.976. Overall, all the classifiers demonstrate similar performance with minor differences, except for the LR classifier which shows slightly lower performance with a Cohen Kappa score of 0.647.

#### Performance on the filtered LMCH data dynamics with 70:30 partition

The SVM classifier exhibits better performance than the LR classifier, except for the Precision metric, where the LR classifier achieves a value of 0.935 compared to 0.929 for the SVM classifier. The performance discrepancy between the KNN classifier and the SVM or LR classifiers is noticeable, with the KNN classifier performing significantly lower. The superiority of the SVM classifier and the lower performance of the KNN classifier are also depicted in the ROC curve shown in Fig 6.

#### Performance on the filtered LMCH data dynamics with 80:20 partition

In the case of diabetes mellitus prediction on the filtered LMCH data dynamics with an 80:20 partition, the SVM classifier outperforms other classifiers for all the performance metrics. The KNN classifier shows lower performance than other classifiers in terms of all performance metrics, except for the AUC value of 0.973, which is greater than the 0.963 AUC of the LR classifier. This is further supported by the ROC curves of the three classifiers shown in Fig 7. Although the performance of the KNN, SVM, and LR classifiers is satisfactory, there is still room for improvement in the classification performance. To address this, we employ filter (IG), wrapper (BFE and SFS), and embedded (RF) feature selection methods.

### Analysis on applying hyperparameter optimization and feature selection based dimensionality reduction

The feature importance according to the filter method and embedded method is depicted in Figs 40–47. We consider the top 4, 5, 7, and 9 features according to filter (IG), wrapper (SFS and BFE), and embedded (RF) methods to train the ML classifiers.

**Fig 40:**
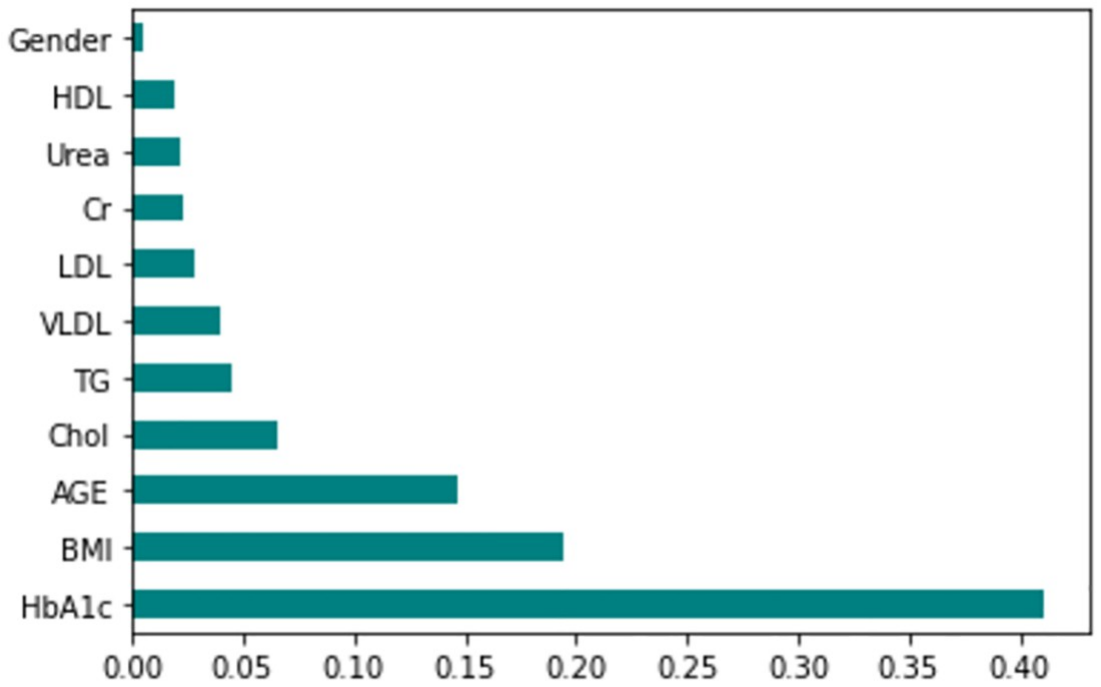
Feature importance based on original LMCH 70:30 with RF.

**Fig 41:**
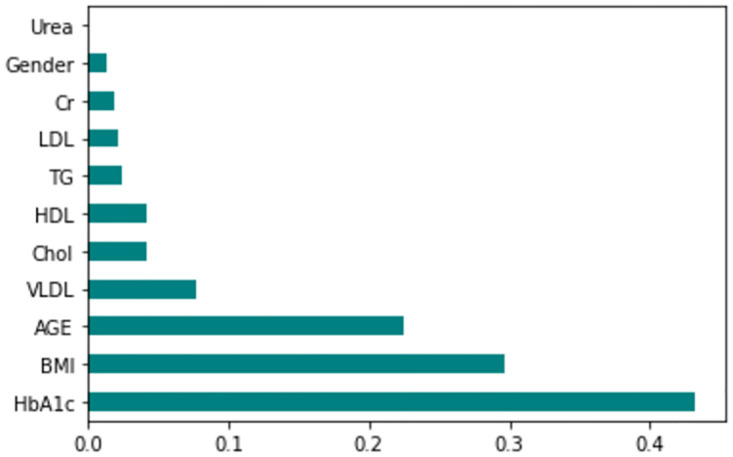
Feature importance based on original LMCH 70:30 with IG.

**Fig 42:**
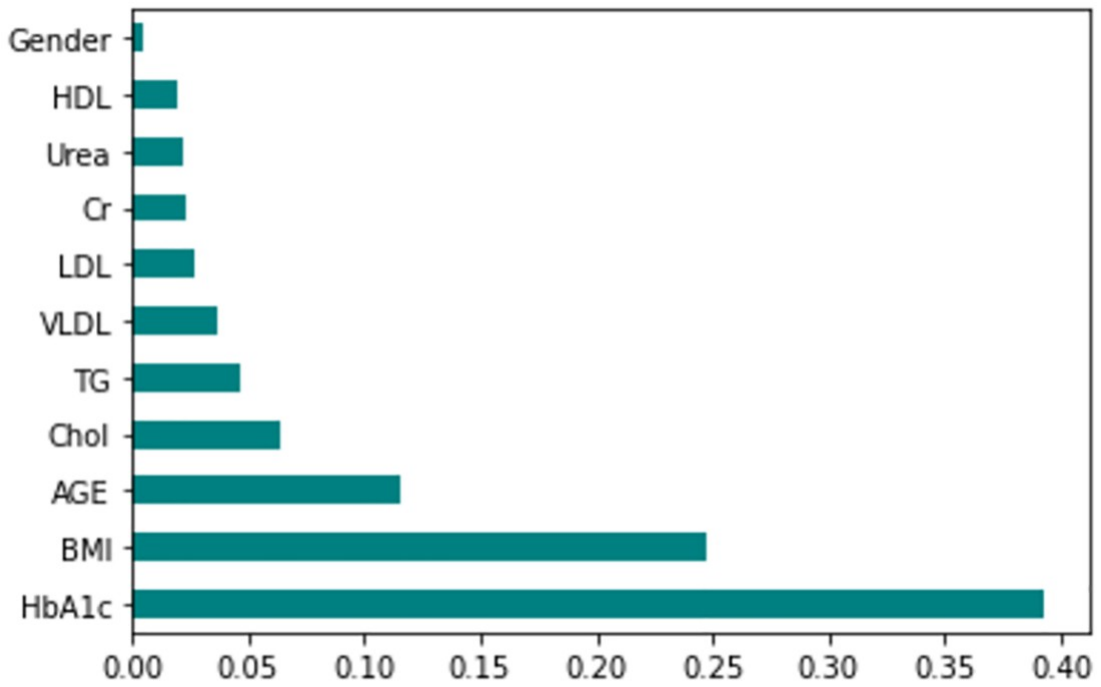
Feature importance based on original LMCH 80:20 with RF.

**Fig 43:**
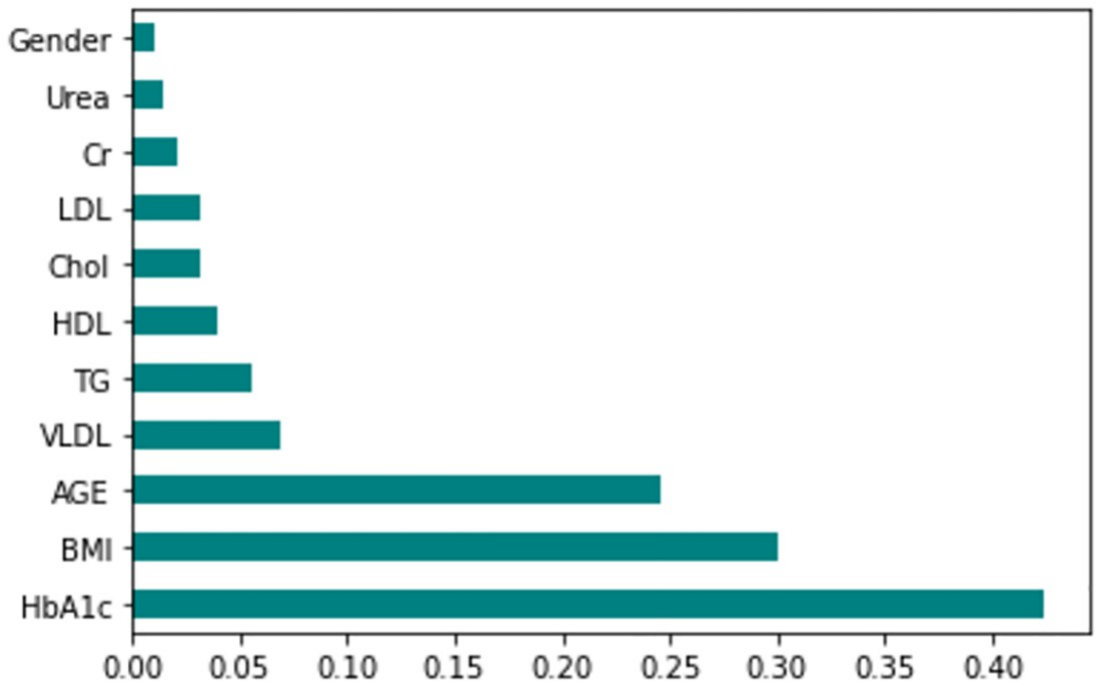
Feature importance based on original LMCH 80:20 with IG.

**Fig 44:**
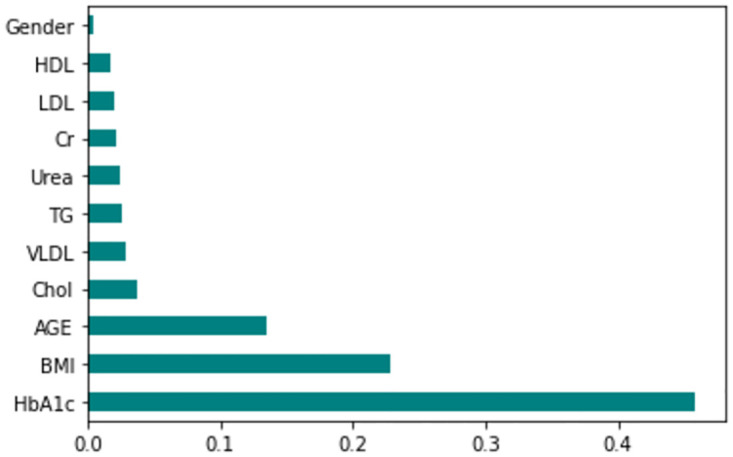
Feature importance based on filtered LMCH 70:30 with RF.

**Fig 45:**
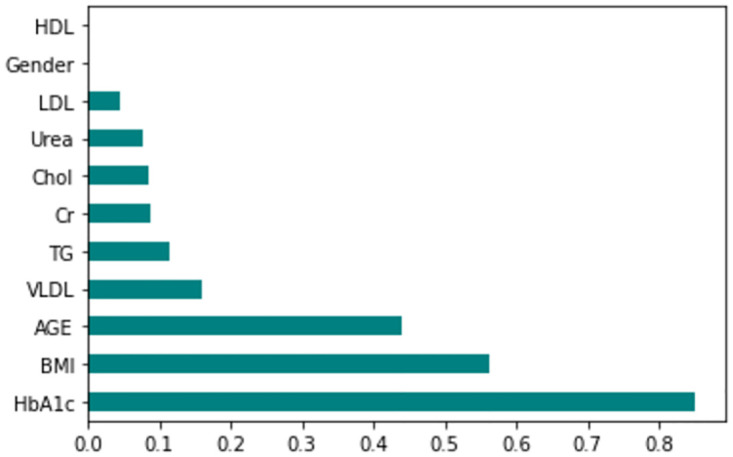
Feature importance based on filtered LMCH 70:30 with IG.

**Fig 46:**
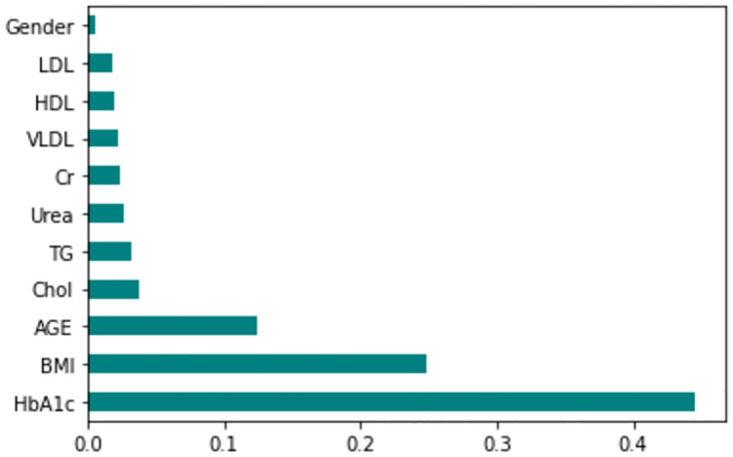
Feature importance based on filtered LMCH 80:20 with RF.

**Fig 47:**
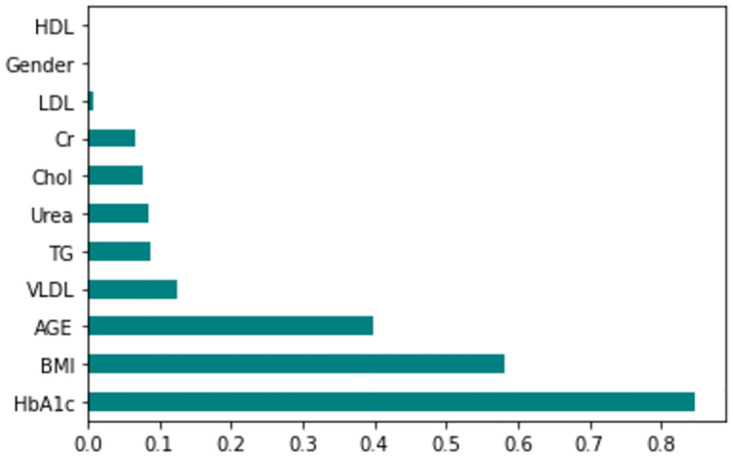
Feature importance based on filtered LMCH 80:20 with IG.

#### Performance on the original extremely imbalanced LMCH data dynamics

Tables 8 and 9 represents the performance of different classifiers by employing features ranked by different feature selection methods for the 70:30 and 80:20 partitions consecutively of the data dynamics.

*70:30 data partition with filter method for feature selection*. The SVM classifier achieved an accuracy of 0.964, precision of 0.968, recall of 0.964, F1-score of 0.962, Cohen’s Kappa of 0.835, and AUC of 0.99 using the top 4 features selected by the IG technique. This outperformed the results produced by using the top 5, 7, and 9 features. Additionally, it outperformed the classification performance of the SVM classifier using all 11 features which means without applying feature selection strategy by 3% accuracy, 4% Cohen’s Kappa, and 4% AUC. The LR classifier provided similar classification performance when using the top 4 and 5 features, as well as when using the top 7 and 9 features. The highest classification performance was achieved using the top 4 and 5 features, which was slightly higher than the performance achieved using the top 7 and 9 features. The SVM classifier achieved the highest results of 0.956 accuracies, 0.955 precision, 0.956 recall, 0.953 F1-score, and 0.798 Cohen’s Kappa using the top 4 features. In terms of the AUC metric, the KNN classifier showed the highest result of 0.985 using the top 7 features. Overall, the SVM classifier outperformed the LR and KNN classifiers for all selected features, considering all the performance metrics. The LR classifier performed slightly lower than the SVM and KNN classifiers, but it outperformed the KNN classifier for some evaluation metrics.

*70:30 data partition with wrapper method for feature selection*. In terms of the BFE and SFS wrapper methods for feature selection, the SFS technique helps achieving better classification performance for all the selected features using the SVM classifier. The SVM classifier using the top 9 selected features by the SFS technique provides an accuracy of 0.942, precision of 0.972, recall of 0.97, F1-score of 0.966, Cohen’s Kappa of 0.908, and AUC of 0.998 which exhibits higher performance than without applying feature selection techniques. The recall score of 0.97 is the highest among the selected features using all the feature selection techniques for the SVM classifier. From Table 8, it is clear that the classification performance increases with more features selected using the SFS wrapper method.

The LR classifier shows advanced classification performance for the selected features using the SFS technique compared to the BFE technique. It is also evident that increasing the number of features in SFS provides increased performance, except for the recall metric for the LR classifier.

Using the selected features according to the SFS technique, the KNN classifier provides the maximum classification performance. The KNN classifier outperforms other classifiers using the selected features of the SFS wrapper method, as shown in Table 8.

*70:30 data partition with an embedded method for feature selection*. The SVM classifier with the top 4 selected features employing the RF embedded method provides the highest result for all the evaluation metrics except accuracy compared to the top 5, 7, and 9 features. As well as this result also outperformed the classification performance obtained without applying feature selection methods. The SVM classifier also shows almost similar classification performance when using the top 4 and 5 features. The LR classifier exhibits somewhat lower performance compared to the SVM and KNN classifiers. In the case of the KNN classifier, the top 4 selected features help achieve the maximum performance. The KNN classifier with the top 4 features provides the highest classification performance among all the features for all the evaluation metrics except accuracy, although this result is closely similar to the classification performance of the SVM classifier with the top 4 features.

*Performance improvement by employing dimensionality reduction compared to the performance achieved without employing feature selection in case of 70:30 data partition*. By employing different feature selection techniques, it can be clearly observed from Table 8 that the classification performance increases by reducing the dimensionality of the feature set.

For the SVM classifier, the performance improves by 1% in accuracy, precision, recall, F1-score, 11% in Cohen Kappa, and 5% in AUC when using the top 9 features selected by the wrapper-based SFS technique. The SVM classifier also demonstrates an improvement of 3% in accuracy, 1% in precision, recall, F1-score, 4% in Cohen Kappa, and 4% in AUC when using the top 4 features selected by the filter-based IG technique. The LR classifier shows an improvement of 3% in accuracy, 2% in precision, 2% in Cohen Kappa, and 1% in AUC by employing the top 4 and 5 features selected by the filter-based IG technique. In terms of the state-of-the-art KNN classifier, the classification performance improves by 1% in accuracy, 4% in precision, recall, F1-score, 20% in Cohen Kappa, and 2% in AUC by using Wrapper-based SFS technique. The KNN classifier demonstrates a 3% improvement in accuracy using the top 4 features selected by the filter-based IG technique. Additionally, the KNN classifier shows improvements of 1% in accuracy, precision, recall, F1-score, 7% in Cohen Kappa, and 1% in AUC by selecting the top 4 features using the embedded RF technique.

*80:20 data partition with filter method for FS*. From Table 9, the SVM classifier achieves an accuracy of 0.946, precision of 0.964, recall of 0.946, F1-score of 0.945, Cohen Kappa of 0.761, and AUC score of 0.99 by using the top 4 and 5 features which exhibits performance improvement compared to performance achieved by without applying feature selection. The LR classifier provides a maximum accuracy of 0.922, precision of 0.942, recall of 0.922, F1-score of 0.918, Cohen Kappa of 0.647, and AUC score of 0.98 by employing the top 4 features. In the case of the KNN classifier, the highest evaluation results are accuracy of 0.958, precision of 0.973, recall of 0.958, F1-score of 0.962, Cohen Kappa of 0.822, and AUC score of 0.99 by selecting the top 7 features.

*80:20 data partition with wrapper method for FS*. The SFS technique helps to achieve better classification performance than the BFE technique as well as without applying feature selection strategy. By using the top 9 features according to the SFS technique, the SVM classifier achieves an accuracy of 0.942, precision of 0.974, recall of 0.972, F1-score of 0.97, Cohen Kappa of 0.911, and AUC of 0.998. In the case of the SFS method, it is clearly observable that increasing the number of features improves the classification performance. The LR classifier shows a maximum accuracy of 0.927, precision of 0.941, recall of 0.935, F1-score of 0.921, Cohen Kappa of 0.789, and AUC score of 0.977 by employing the top 9 features according to the BFE technique. In the case of the KNN classifier, an accuracy of 0.935 and precision, recall, F1-score, Cohen Kappa, and AUC score of 1.0 are achieved by using the top 9 features according to the SFS technique.

*80:20 data partition with an embedded method for FS*. The SVM classifier achieved the highest accuracy of 0.95, the precision of 0.972, recall of 0.964, F1-score of 0.966, Cohen Kappa of 0.841, and AUC of 0.978 by employing the top 4 features which outperformed the result obtained by without utilizing feature selection strategy. The LR classifier shows similar results for the top 4 and 5 features and also achieves the highest classification performance for some evaluation metrics by employing the top 7 and 9 features. The KNN classifier shows the highest classification performance for all the evaluation metrics except accuracy when selecting the top 4 features.

**Performance improvement by employing dimensionality reduction compared to the performance achieved without employing feature selection** in case of 80:20 data partition. For the SVM classifier, selecting the top 4 features according to the embedded-based RF techniques led to a 1% improvement in accuracy, 2% improvement in precision, 2% improvement in recall, 3% improvement in F1-score, 12% improvement in Cohen Kappa, and 2% improvement in AUC score. Furthermore, selecting the top 9 features using the wrapper-based SFS method resulted in a 2% improvement in precision, 3% improvement in recall, 3% improvement in F1-score, 19% improvement in Cohen Kappa, and 4% improvement in AUC score. The LR classifier showed the maximum improvement of 1% in accuracy, 2% in the recall, 14% in Cohen Kappa, and 1% in the AUC score by employing the top 9 features selected by BFE. Similarly, the KNN classifier exhibited performance improvements of 4% in precision, 5% in the recall, 5% in F1-score, 23% in Cohen Kappa, and 2% in AUC score by using the top 9 features selected by SFS. The optimized hyperparameter for the classifiers is tabulated in Tables 12 and 13.

#### Performance on the filtered moderately imbalanced LMCH data dynamics

Tables 10 and 11 presents the performance of different classifiers by employing features ranked using different feature selection methods for the 70:30 and 80:20 partitions consecutively of the data dynamics.

*70:30 data partition with filter method for FS*. The SVM classifier with the top 4 selected features achieves a maximum precision of 0.932, recall of 0.925, F1-score of 0.927, Cohen Kappa of 0.878, and AUC of 0.991 than other selected features. In the case of accuracy metric, the performance achieved with the top 4 selected features outperformed the accuracy obtained without utilizing feature selection techniques. The maximum accuracy of 0.968 is achieved with the top 5 features. The ROC plot in Fig 8 also visually demonstrates the superior classification performance of the SVM classifier with the top 4 features. The LR classifier achieves a maximum accuracy of 0.891, precision of 0.938, recall of 0.938, F1-score of 0.938, Cohen Kappa of 0.897, and AUC of 0.972 with a minimum of top 4 features. The KNN classifier achieves the highest accuracy of 0.946 with the top 5 features. The KNN classifier shows the highest classification performance for all evaluation metrics except accuracy when selecting the top 7 features. The ROC plots in Figs 8–11 clearly visualize the highest classification performance when using top 4 selected features, although the results for top 4 and top 5 features are mostly similar. The ROC plots also demonstrate the superiority of the SVM classifier.

*70:30 data partition with wrapper method for FS*. The SVM classifier achieves the highest accuracy of 0.912, precision of 0.976, recall of 0.975, F1-score of 0.975, Cohen Kappa of 0.959, and AUC of 0.997 by utilizing the top 5 features selected by BFE techniques, and this result also outperformed the classification performance obtained without applying feature selection techniques. The SVM classifier also provides a maximum accuracy of 0.923, precision of 0.988, recall of 0.988, F1-score of 0.987, Cohen Kappa of 0.979, and AUC of 0.999 by selecting the top 7 features according to SFS techniques. The LR classifier achieves a maximum accuracy of 0.874, precision of 0.945, recall of 0.938, F1-score of 0.934, Cohen Kappa of 0.894, and AUC of 0.992 by choosing the top 4 and 5 features according to SFS techniques. The KNN classifier achieves a maximum accuracy of 0.868, precision of 0.938, recall of 0.938, F1-score of 0.937, Cohen Kappa of 0.896, and AUC of 0.987 for the top 4 and 5 features provided by SFS techniques. The ROC plot in Fig 27 demonstrates the best classification performance of the SVM classifier using the top 9 selected features among the ROC plots of SFS and BFE techniques-based selected features.

*70:30 data partition with an embedded method for FS*. The state-of-the-art SVM classifier achieves a maximum precision of 0.932, recall of 0.925, F1-score of 0.927, Cohen Kappa of 0.878, and AUC of 0.991 by selecting the top 4 features. In terms of accuracy, the SVM classifier achieves the highest score of 0.968 by using the top 5 features. The LR classifier also shows comparatively better classification performance with an accuracy of 0.891, precision of 0.938, recall of 0.938, F1-score of 0.938, Cohen Kappa of 0.897, and AUC of 0.972 for the top 4 features. On the other hand, the KNN classifier achieves the highest classification performance with a precision of 0.927, recall of 0.925, F1-score of 0.923, Cohen Kappa of 0.875, and AUC of 0.973 by selecting the top 7 features. The ROC plots in Figs 32 and 33 demonstrate the robustness of the SVM classifier for the top 4 and 5 features.

**Performance improvement by employing dimensionality reduction compared to the performance achieved without employing feature selection** in case of 70:30 data partition:

In the case of the SVM classifier, the evaluation metrics show a performance improvement of 5% in accuracy and 1% in AUC by selecting the top 4 features using the filter-based IG and embedded-based RF techniques. By considering the top 7 features according to the SFS algorithm, the SVM classifier achieves a performance improvement of 3% in accuracy, 6% in precision, 6% in the recall, 6% in F1-score, 10% in Cohen Kappa, and 2% in AUC. The LR classifier achieves a performance improvement of 2% in accuracy, 1% in recall, 1% in F1-score, and 2% in Cohen Kappa by selecting the top 4 features using the filter-based IG and embedded-based RF methods. The KNN classifier shows a significant improvement of 7% in accuracy, 10% in precision, 12% in recall, 10% in F1-score, 18% in Cohen Kappa, and 5% in AUC by selecting the top 7 features using the embedded-based RF algorithm.

**80:20 data partition with filter method for FS**. The SVM classifier achieves the highest accuracy of 0.957 by selecting the top 4 features. It also achieves the highest precision of 0.948, recall of 0.943, F1-score of 0.945, Cohen Kappa of 0.909, and AUC of 0.989 for the top 7 features. The LR classifier achieves an accuracy of 0.919, precision of 0.944, recall of 0.943, F1-score of 0.942, Cohen Kappa of 0.907, and AUC of 0.987 by selecting the top 4 features. The KNN classifier shows the highest accuracy of 0.944 by selecting the top 4 features. It also achieves the highest precision of 0.948, recall of 0.943, F1-score of 0.945, Cohen Kappa of 0.908, and AUC of 0.988 by selecting the top 5 features. The ROC curves in Figs 12, 13 and 15 clearly show the superior performance of the SVM classifier using the top 4, 5, and 9 features, respectively.

**80:20 data partition with wrapper method for FS**. The SVM classifier achieves a maximum accuracy of 0.938, and all other performance metrics reach 1.0. The LR classifier achieves the highest accuracy of 0.914, precision of 0.982, recall of 0.981, F1-score of 0.981, Cohen Kappa of 0.969, and AUC of 0.998 by selecting the top 4 features using BFE and SFS techniques. The KNN classifier also achieves a maximum accuracy of 0.914, precision of 0.959, recall of 0.943, F1-score of 0.946, Cohen Kappa of 0.91, and AUC of 0.997 by selecting the top 4 features using BFE and SFS feature selection techniques.

Based on the ROC curves in Figs 21–23, 29 and 30, it can be observed that the LR classifier performs the best. However, the ROC curves in Figs 20 and 28 show that the KNN classifier performs better than the others when using the top 4 features selected by BFE and SFS techniques, respectively.

**80:20 data partition with the embedded method for FS**. The SVM classifier achieves a comparatively better precision of 0.947, recall of 0.943, F1-score of 0.944, Cohen Kappa of 0.908, and AUC of 0.996 by selecting the top 5 features. In terms of accuracy, the SVM achieves a maximum of 0.957 by selecting the top 4 features. The LR classifier achieves a maximum accuracy of 0.914, precision of 0.942, recall of 0.943, F-1 score of 0.942, Cohen Kappa of 0.907, and AUC of 0.968 by selecting the top 5 features. The state-of-the-art KNN classifier achieves an accuracy of 0.939, precision of 0.924, recall of 0.925, F-1 score of 0.923, Cohen Kappa of 0.876, and AUC of 0.984 by selecting the top 4 features. Based on the ROC curves in Figs 36, 37 and 39, it can be observed that the SVM classifier outperforms the others for the top 4, 5, and 9 features, respectively.

**Performance improvement by employing dimensionality reduction compared to the performance achieved without employing feature selection** in case of 80:20 data partition. The state-of-the-art SVM classifier shows an improvement of 4% accuracy, 6% precision, 6% recall, 6% F1-score, 9% Cohen Kappa, and 1% AUC score by selecting the top 4 features using the wrapper-based SFS method. The LR classifier shows an improvement of 2% accuracy, 7% precision, 7% recall, 7% F1-score, 12% Cohen Kappa, and 4% AUC score by selecting the top 4 features according to the wrapper-based SFS method. In the case of the KNN classifier, the maximum performance improvement is about 5% accuracy, 7% precision, 5% recall, 7% F1-score, 10% Cohen Kappa, and 3% AUC by selecting the top 4 features using the wrapper-based BFE and SFS techniques. By analyzing the evaluation metrics described above, it is clear that the SVM classifier performs comparatively better when selecting a small number of features, specifically 4 and 5.

### Effectiveness measure of feature selection procedure via scatter plot visualization of features

**Fig 48:**
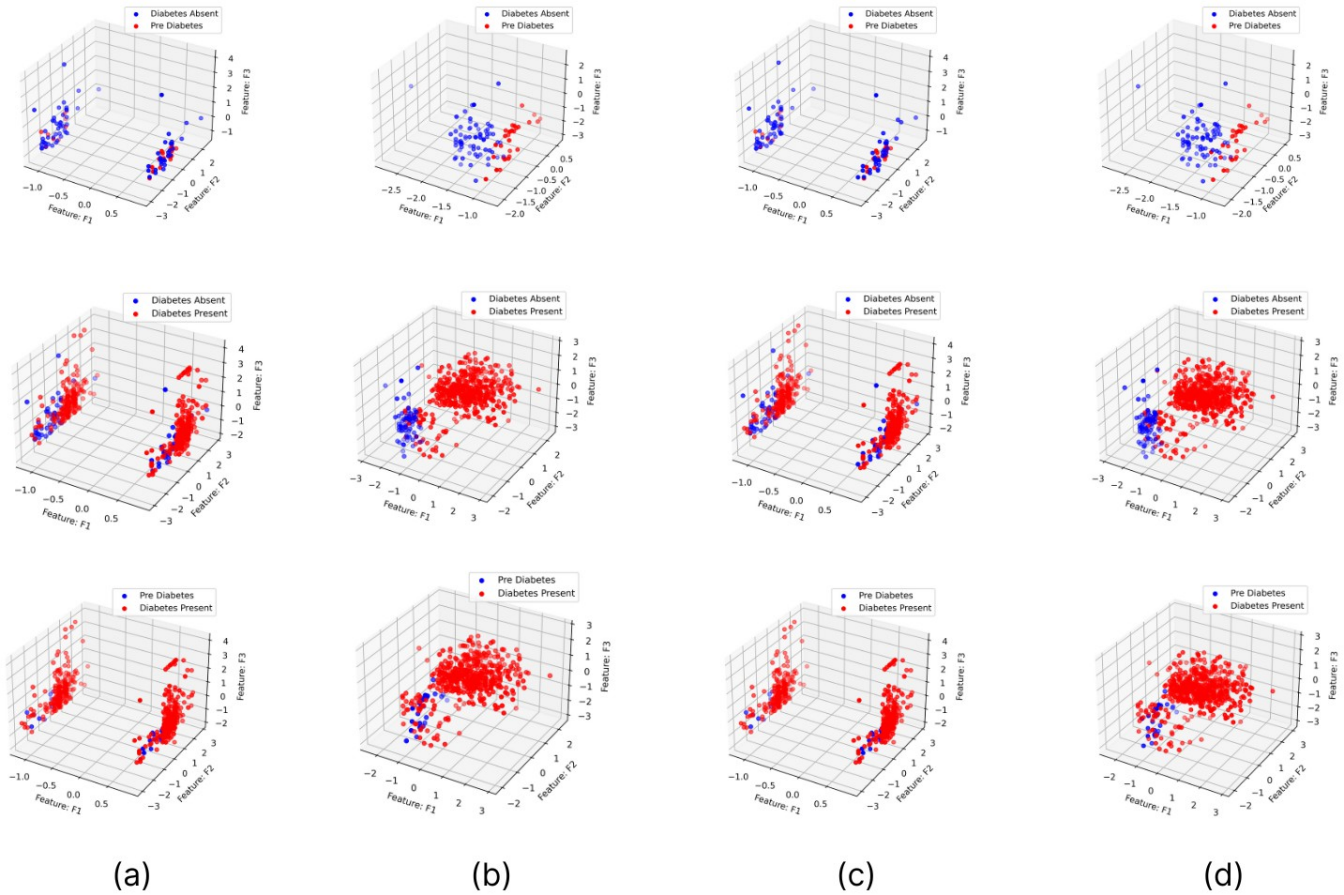
Scatter plot of (a) First three features without feature selection based on extremely imbalanced data with 70:30 partition (b) First three features with feature selection based on extremely imbalanced data with 70:30 partition (c) First three features without feature selection based on extremely imbalanced data with 80:20 partition (d) First three features with feature selection based on extremely imbalanced data with 80:20 partition.

**Fig 49:**
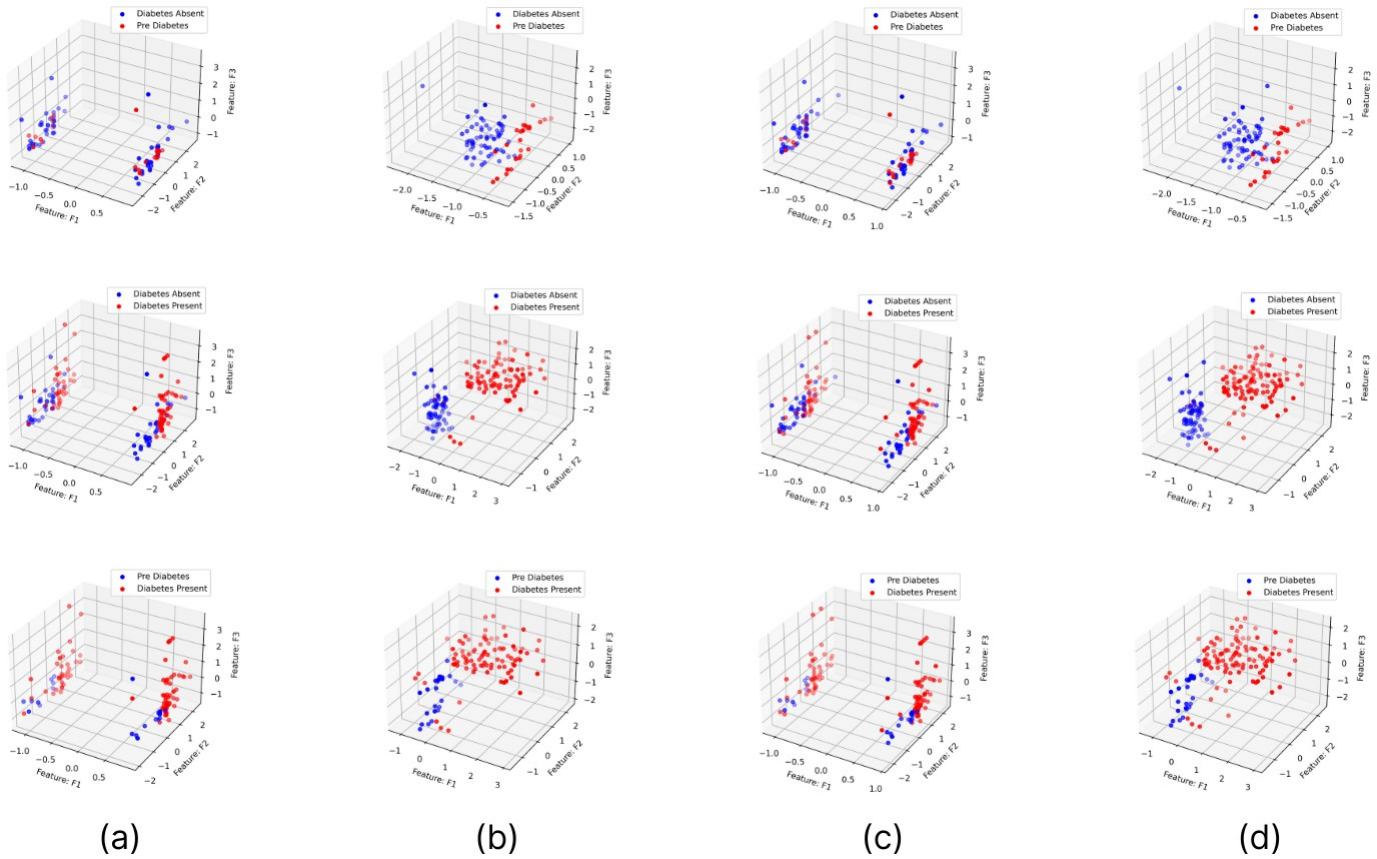
Scatter plot of (a) First three features without feature selection based on moderately imbalanced data with 70:30 partition (b) First three features with feature selection based on moderately imbalanced data with 70:30 partition (c) First three features without feature selection based on moderately imbalanced data with 80:20 partition (d) First three features with feature selection based on moderately imbalanced data with 80:20 partition.

If features are distinctly separated, it becomes easier to determine their respective classes [60]. According to the embedded-based RF feature selection procedure, scatter plots of the top three features based on extremely imbalanced data with a 70:30 partition are visualized in Fig 48b, depicting better class separability than the first three features without feature ranking in Fig 48a. Additionally, in the case of extremely imbalanced data with an 80:20 partition, the scatter plot visualizations in Fig 48d of the top three features according to the RF approach show better class separability compared to those without feature selection in Fig 48c. In the case of our proposed moderately imbalanced dataset with 70:30 and 80:20 partitions, the top 3 three features identified using the RF technique exhibit better feature separability. This is visualized in Fig 49b and 49d, respectively, compared to the visualization of the top three features without feature selection in Fig 49a and 49b.

### Performance comparison with previous research utilizing a similar LMCH dataset

Table 16 presents a comparison of our work with state-of-the-art research. It demonstrates that our work consistently outperforms the previous research in terms of precision, recall, and F1-score. While we have not listed all the experiments where our work outperforms the existing research in this table, the overall trend indicates superior performance in these evaluation metrics.

**Table 16:** Comparison table showcasing our proposed research performance with respect to previous research that was conducted based on the same LMCH dataset.

Ref.	Dataset Partition	Dataset	FS Algo.	Sel. Fea.	Model	Acc.	Pre.	Rec.	F1 Sc.	C.K.	AUC
[10]	Extremely Imbalance LMCH	-	Polynomial Regression	-	2GDNN	97.33%	97.25%	97.33%	97.27%	-	-
[17]	Randomly selected 392 records from LMCH	Randomly selected 392 records	-	-	AOWD	98.95%	98.88%	-	-	-	-
Our Pro- posed	Extremely Imbalance LMCH	70:30, 80:20	SFS	9	KNN	94%	100%	100%	100%	100%	100%
		70:30, 80:20	SFS	5, 7	KNN	92%	100%	100%	100%	100%	100%
		80:20	IG	7	KNN	96%	97%	96%	96%	82%	99%
		70:30	IG	4	SVM	96.40%	97%	96%	96%	84%	99%
		80:20	RF	4	SVM	95%	97%	96.40%	97%	84%	98%
	Our Created Moderately Imbalance LMCH	80:20	SFS	5	SVM	92.90%	100%	100%	100%	100%	100%
		80:20	BFE, SFS	4	SVM	94%	100%	100%	100%	100%	100%
		80:20	BFE, SFS	4	LR	91.40%	98%	98%	98%	97%	100%

They Python code of this work is available in the GitHub repository [61].

## Conclusion and future work

Multiclass diabetes mellitus classification does not have access to a public balanced dataset (where the ratio of classes is same) or moderately balanced dataset (with a class ratio of 40:60). However, the performance and reliability of machine learning models depend on the characteristics of the data. It is well known that balanced or moderately balanced datasets are more reliable and provide better predictive performance than extremely imbalanced datasets. This paper primarily focuses on the generation of a new moderately imbalanced LMCH dataset from the existing extremely imbalanced LMCH dataset. Also, rigorous and empirical statistical analyses for multiclass diabetes classification are performed. Moreover, various feature selection methods based on dimensionality reduction techniques were applied to reduce the data dimension. Among the classifiers investigated, the KNN classifier consistently showed better overall classification performance in the experiments. The SVM classifier performed well and achieved the second-best overall classification performance in several experiments. The LR classifier showed slightly lower performance compared to the SVM and KNN classifiers, but in some experiments, it outperformed them. The high classification or detection accuracy achieved by the different classifiers demonstrates the effectiveness of our applied preprocessing pipeline for biomedical research. The creation of the filtered moderately imbalanced LMCH dataset yielded reliable results as it reduced the instance discrepancy between multiple classes compared to the original extremely imbalanced LMCH data dynamics. The limitation of our proposed methodology is that we utilized traditional machine learning models, which are unable to provide data confidentiality since they aggregate all the training data in one place before model training.

In future work, one important aspect we will focus on is ensuring user privacy and data confidentiality, which are critical concerns in the present century. To address this issue, we will explore the transition from a traditional machine learning model to a federated learning model for diabetes disease predictions. Federated learning allows us to train models across decentralized devices, keeping data within individual medical centers while ensuring user data remains localized and private. By adopting this approach, we aim to mitigate privacy risks and ensure that sensitive data remains secure throughout the predictive process. Another aim is to explore the adoption of the Shapley Additive Explanations (SHAP) method for measuring feature importance and interpreting the performance of machine learning models. By employing SHAP, we intend to gain a deeper understanding of how various features contribute to the outcomes of our models. Additionally, we plan to investigate and test DL models for diabetes prediction. The DL has shown promise in various fields, and we believe it holds potential for enhancing the accuracy and reliability of diabetes prediction models.
